# Current global status of male reproductive health

**DOI:** 10.1093/hropen/hoae017

**Published:** 2024-04-12

**Authors:** Christopher J De Jonge, Christopher L R Barratt, R John Aitken, Richard A Anderson, Peter Baker, David Y L Chan, Mark P Connolly, Michael L Eisenberg, Nicolas Garrido, Niels Jørgensen, Sarah Kimmins, Csilla Krausz, Robert I McLachlan, Craig Niederberger, Moira K O’Bryan, Allan Pacey, Lærke Priskorn, Satu Rautakallio-Hokkanen, Gamal Serour, Joris A Veltman, Donna L Vogel, Mónica H Vazquez-Levin

**Affiliations:** Department of Urology, University of Minnesota Medical Center, University of Minnesota, Minneapolis, MN, USA; Division of Systems Medicine, School of Medicine, Ninewells Hospital and Medical School, University of Dundee, Dundee, UK; Discipline of Biological Sciences, School of Environment and Life Sciences, College of Engineering, Science and Environment, University of Newcastle, Newcastle, Australia; MRC Centre for Reproductive Health, University of Edinburgh, Edinburgh, UK; Global Action on Men’s Health, UK; Assisted Reproductive Technology Unit, Department of Obstetrics and Gynaecology, Faculty of Medicine, The Chinese University of Hong Kong, Shatin, China; Health Economics, Global Market Access Solutions LLC, Mooresville, NC, USA; University Medical Center Groningen, Groningen, The Netherlands; Department of Urology and Obstetrics & Gynecology, Stanford University, Stanford, CA, USA; IVIRMA Global Research Alliance, IVI Foundation, Instituto de Investigación Sanitaria La Fe (IIS La Fe), Valencia, Spain; Department of Growth and Reproduction and International Center for Research and Research Training in Endocrine Disruption of Male Reproduction and Child Health (EDMaRC), Rigshospitalet, University of Copenhagen, Copenhagen, Denmark; Department of Pharmacology and Therapeutics, Faculty of Medicine, McGill University, Montreal, QC, Canada; Centre de Recherche du Centre Hospitalier de l’Université de Montréal, Montréal, QC, Canada; Département de Pathologie et Biologie Cellulaire, Université de Montréal, Montréal, QC, Canada; Department of Experimental and Clinical Biomedical Sciences, ‘Mario Serio’, University of Florence, University Hospital of Careggi (AOUC), Florence, Italy; Hudson Institute of Medical Research, Monash University, Melbourne, Australia; Monash IVF Group, Cremorne, Australia; Clarence C. Department of Urology, University of Illinois Chicago (UIC), College of Medicine, Department of Bioengineering, UIC College of Engineering, Chicago, IL, USA; School of BioSciences and Bio21 Institute, The University of Melbourne, Parkville, Australia; Faculty of Biology, Medicine and Health, Core Technology Facility, University of Manchester, Manchester, UK; Department of Growth and Reproduction and International Center for Research and Research Training in Endocrine Disruption of Male Reproduction and Child Health (EDMaRC), Rigshospitalet, University of Copenhagen, Copenhagen, Denmark; Fertility Europe, Evere, Belgium; The International Islamic Center for Population Studies and Research, Al-Azhar University, Maadi, Cairo, Egypt; Egyptian IVF Center, Maadi, Cairo, Egypt; Faculty of Medical Sciences, Biosciences Institute, Newcastle University, Newcastle upon Tyne, UK; School of Medicine, Johns Hopkins University, Baltimore, MD, USA; Instituto de Biología y Medicina Experimental, Consejo Nacional de Investigaciones Científicas y Técnicas de Argentina—Fundación IBYME, Buenos Aires, Argentina

**Keywords:** male infertility, male reproductive health, andrology, fertility, contraception, genetics, epigenetics, education, economics, policy

## Abstract

**BACKGROUND:**

The widespread interest in male reproductive health (MRH), fueled by emerging evidence, such as the global decline in sperm counts, has intensified concerns about the status of MRH. Consequently, there is a pressing requirement for a strategic, systematic approach to identifying critical questions, collecting pertinent information, and utilizing these data to develop evidence-based strategies. The methods for addressing these questions and the pathways toward their answers will inevitably vary based on the variations in cultural, geopolitical, and health-related contexts. To address these issues, a conjoint ESHRE and Male Reproductive Health Initiative (MRHI) Campus workshop was convened.

**OBJECTIVE AND RATIONALE:**

The three objectives were: first, to assess the current state of MRH around the world; second, to identify some of the key gaps in knowledge; and, third, to examine how MRH stakeholders can collaboratively generate intelligent and effective paths forward.

**SEARCH METHODS:**

Each expert reviewed and summarized the current literature that was subsequently used to provide a comprehensive overview of challenges related to MRH.

**OUTCOMES:**

This narrative report is an overview of the data, opinions, and arguments presented during the workshop. A number of outcomes are presented and can be summarized by the following overarching themes: MRH is a serious global issue and there is a plethora of gaps in our understanding; there is a need for widespread international collaborative networks to undertake multidisciplinary research into fundamental issues, such as lifestyle/environmental exposure studies, and high-quality clinical trials; and there is an urgent requirement for effective strategies to educate young people and the general public to safeguard and improve MRH across diverse population demographics and resources.

**LIMITATIONS, REASONS FOR CAUTION:**

This was a workshop where worldwide leading experts from a wide range of disciplines presented and discussed the evidence regarding challenges related to MRH. While each expert summarized the current literature and placed it in context, the data in a number of areas are limited and/or sparse. Equally, important areas for consideration may have been missed. Moreover, there are clear gaps in our knowledge base, which makes some conclusions necessarily speculative and warranting of further study.

**WIDER IMPLICATIONS:**

Poor MRH is a global issue that suffers from low awareness among the public, patients, and heathcare professionals. Addressing this will require a coordinated multidisciplinary approach. Addressing the significant number of knowledge gaps will require policy makers prioritizing MRH and its funding.

**STUDY FUNDING/COMPETING INTEREST(S):**

The authors would like to extend their gratitude to ESHRE for providing financial support for the Budapest Campus Workshop, as well as to Microptic S.L. (Barcelona) for kindly sponsoring the workshop. P.B. is the Director of the not-for-profit organization Global Action on Men’s Health and receives fees and expenses for his work, (which includes the preparation of this manuscript). Conflicts of interest: C.J.D.J., C.L.R.B., R.A.A., P.B., M.P.C., M.L.E., N.G., N.J., C.K., AAP, M.K.O., S.R.-H., M.H.V.-L.: ESHRE Campus Workshop 2022 (Travel support—personal). C.J.D.J.: Cambridge University Press (book royalties—personal). ESHRE Annual Meeting 2022 and Yale University Panel Meeting 2023 (Travel support—personal). C.L.R.B.: Ferring and IBSA (Lecture), RBMO editor (Honorarium to support travel, etc.), ExSeed and ExScentia (University of Dundee), Bill & Melinda Gates Foundation (for research on contraception). M.P.C.: Previously received funding from pharmaceutical companies for health economic research. The funding was not in relation to this work and had no bearing on the contents of this work. No funding from other sources has been provided in relation to this work (funding was provided to his company Global Market Access Solutions). M.L.E.: Advisor to Ro, Doveras, Next, Hannah, Sandstone. C.K.: European Academy of Andrology (Past president UNPAID), S.K.: CEO of His Turn, a male fertility Diagnostic and Therapeutic company (No payments or profits to date). R.I.M.: www.healthymale.org.au (Australian Government funded not for profit in men’s health sector (Employed as Medical Director 0.2 FET), Monash IVF Pty Ltd (Equity holder)). N.J.: Merck (consulting fees), Gedeon Richter (honoraria). S.R.-H.: ESHRE (Travel reimbursements). C.N.: LLC (Nursing educator); COMMIT (Core Outcomes Measures for Infertility Trials) Advisor, meeting attendee, and co‐author; COMMA (Core Outcomes in Menopause) Meeting attendee, and co‐author; International Federation of Gynecology and Obstetrics (FIGO) Delegate Letters and Sciences; ReproNovo, Advisory board; American Board of Urology Examiner; American Urological Association Journal subsection editor, committee member, guidelines co‐author Ferring Scientific trial NexHand Chief Technology Officer, stock ownership Posterity Health Board member, stock ownership. A.P.: Economic and Social Research Council (A collaborator on research grant number ES/W001381/1). Member of an advisory committee for Merck Serono (November 2022), Member of an advisory board for Exceed Health, Speaker fees for educational events organized by Mealis Group; Chairman of the Cryos External Scientific Advisory Committee: All fees associated with this are paid to his former employer The University of Sheffield. Trustee of the Progress Educational Trust (Unpaid). M.K.O.: National Health and Medical Research Council and Australian Research Council (Funding for research of the topic of male fertility), Bill and Melinda Gates Foundation (Funding aimed at the development of male gamete-based contraception), Medical Research Future Fund (Funding aimed at defining the long-term consequences of male infertility). M.H.V.-L.: Department of Sexual and Reproductive Health and Research (SRH)/Human Reproduction Programme (HRP) Research Project Panel RP2/WHO Review Member; MRHI (Core Group Member), COMMIT (member), EGOI (Member); Human Reproduction (Associate Editor), Fertility and Sterility (Editor), AndroLATAM (Founder and Coordinator).

WHAT DOES THIS MEAN FOR PATIENTS? There is a growing interest in men’s reproductive health because of new evidence showing a decline in sperm counts worldwide. Researchers have found links between poor reproductive health in men and other health problems. They are also looking into how a father’s health can affect their children’s well-being. To tackle these issues, the European Society for Human Reproduction and Embryology and the Male Reproductive Health Initiative organized an international workshop. The goals were to assess the current state of men’s reproductive health globally, pinpoint knowledge gaps, and come up with plans for the future. This report summarizes the information, opinions, and discussions from the workshop. The main takeaways are that men’s reproductive health is a serious global concern and there is a lot we still do not understand. The report emphasizes the need for international collaborations to study important issues like the impact of lifestyle and environmental factors. It also highlights the urgency of finding effective ways to reinforce education about how to protect and improve men’s reproductive health across different demographics (for example age, race) and resources. To complement this analysis, we have recently published a practical plan, based on the evidence, to guide us in moving forward. This plan emphasizes the importance of everyone around the world working together to make men’s reproductive health a top priority.

## Introduction

There is a surge of interest in male reproductive health (MRH) fueled by emerging evidence of globally declining sperm counts (Levine *et al.*, 2023), associations between poor MRH and somatic disorders (Latif *et al.*, 2017; Belladelli *et al.*, 2023), and the impact of paternal morbidities on the next generation (Yu *et al.*, 2023), among others. As with any emerging health concern, there is a need for a rational approach to identify the important questions, gather intelligence, and use the data to formulate evidence-based actions (Kimmins *et al.*, 2024).

With the aforementioned as a brief background, a conjoined ESHRE (Supplementary Table S1 provides URLs list of all organizations mentioned in the report) and MRH Initiative (MRHI) Campus Workshop (Supplementary Data File S1) was convened to examine the current state of MRH around the world, to articulate some of the overarching challenges, and examine how MRH stakeholders can collaboratively generate effective paths forward. This narrative report presents an overview of the data, opinions, and arguments presented during the meeting. The information presented has been organized in three main sections: MHR: a global perspective; MRH: science & medicine; and MRH: society, economics, policies & education. To finalize, the report brings conclusions for a way forward.

## MRH: a global perspective

### Sustainable development goals and MRH

In 2015, the United Nations (UN) produced a list of 17 sustainable development goals (SDGs) (Supplementary Data File S2), aimed to end extreme poverty, give people better healthcare, achieve equality for women, protect the planet, and ensure prosperity for everyone by the year 2030. The scope of MRH extends across a wide spectrum of SDGs, encompassing Goals 1 to 5, 8, 10, and 13. Addressing MRH contributes to the achievement of reproductive health rights and other human rights, reducing inequality, empowering women and adolescents, improving human capital and health, mitigating adverse environmental and climate impacts, and preventing catastrophic health expenditures.

The World Health Organization (WHO) defines sexual health as ‘a state of physical, emotional, mental, and social well-being in relation to sexuality…not merely the absence of disease, dysfunction or infirmity’ (Supplementary Table S2). Sexual health is part of the overall health and well-being agenda and is, as outlined below, closely tied to SDG 3 and 5 with strong links to other health conditions such as diabetes mellitus, cardiovascular disease, and depression. Thus, these interwoven goals reinforce the importance of taking a broad holistic approach to men’s sexual health and well-being.

Men’s access to fertility care is very relevant to SDGs 1, 3, 5, and 10 because of barriers that create inequitable access, particularly for the poor, unmarried, under-educated, unemployed, and marginalized (SDG 10). Fertility care is expensive, and more so in low/middle-income countries where it is rarely prioritized, leading to unsustainable health expenditures (SDG 1). Among infertile couples, irrespective of the cause of infertility, gender-based violence, social stigma, depression, anxiety, and low self-esteem are more common than in fertile couples (SDG 5). Fear of infertility can deter women and men from using contraception and failure to provide fertility care denies affected individuals of their right to decide the number, timing, and spacing of children (SDG 3) (WHO, 2023a).

Male contraception is critical to equity when addressing family planning, and a lack of modern methods for male contraception (see ‘New methods of male-based contraception’ section) contributes to unmet SDG 3 and SDG indicator 3.7.1. The necessary development of a greater selection, availability, and use of male contraception would lessen the burden on women for contraception, thus leading to greater equity, allow for proactively spacing pregnancies, prevent unintended pregnancies and unsafe abortions, and address multiple SDGs. Some examples include disrupting the cycle of poverty (SDG 1), reducing food demand (SDG 2), improving maternal and child health (SDG 3), increasing school attendance and completion (SDG 4), facilitating greater employment and education for women and girls (SDG 5), facilitating greater workforce participation by women (SDG 8), reducing inequalities (SDG 10), and reducing population pressure on the environment (SDG 13).

Suffice it to say, aspects of MRH, sexual health and well-being, contraception, and infertility, are important but frequently neglected issues which, if addressed, would support achievement of several SDGs. The WHO has substantial resources that can be used as a starting point to better address MRH (Supplementary Table S2).

### Population dynamics

Fertility rates are closely tied to prosperity (Aitken, 2022a). As nations ascend the socioeconomic scale, family sizes decline in a process known as the demographic transition. The link between falling fertility rates and the demographic transition is complex, but at least five major factors may be identified:

- as nations become more socioeconomically developed, primary healthcare improves, and infant as well as childhood mortality rates decline;
- a modern industrialized society is associated with fertility rates decline in association with increased availability of contraception and costs of housing and child raising;
- an increase in the rates of female and male education and a delay initiating a family until their professional goals have been achieved and their career security is assured;
- increased industrialization means increased exposure to environmental pollutants including common reproductive toxicants, as well as to radiofrequency electromagnetic radiation;
- the decreased selection pressure on high-fertility genes and the exponential growth of the assisted conception industry may serve to keep poor fertility genes within the population (Madsen *et al.*, 2018; Aitken, 2022a,b; Fauser *et al.*, 2024).

The decline in the number of children per female is a multifaceted phenomenon, extending beyond merely an increase in infertility rates. It involves a delicate interplay between personal choices and biological factors, underscoring the complexity of the transition. This shift, influenced in part by economic considerations at the individual and couple levels, also signals broader implications for national economies. The intricate dynamics of choice versus biology in the context of declining fertility rates highlight the far-reaching impact of this trend on both personal decisions and the socio-economic landscape at a national scale.

**Figure 1.**
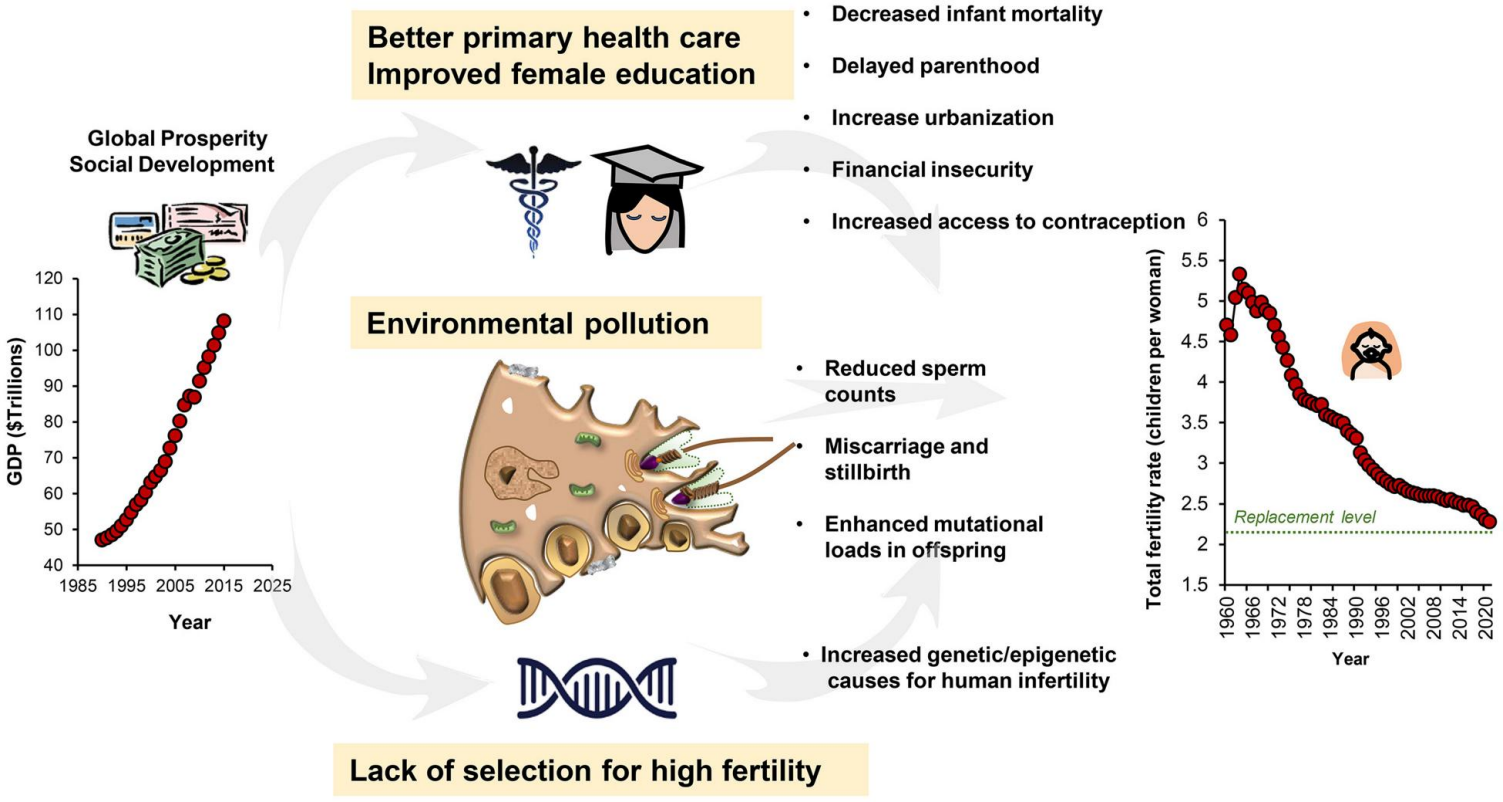
**The impact of global prosperity on human fertility.** As nations become more socio-economically developed, primary healthcare improves, infant mortality rates decline and family sizes decrease. In parallel, women use the freedom they have gained from the reduction in family size to become educated and pursue a professional career. This frequently leads to a delay in initiating a family, which further compromises fertility owing to advanced maternal age. In parallel, the increased urbanization associated with modern industrialized societies further challenges our fertility, as a result of the increased availability of contraception, the decline in available living space and the increased cost of housing and child raising. In the longer term, the environmental pollution associated with urban living impacts fertility, reducing sperm counts and increasing the risk of miscarriage and stillbirth. Such pollutants, as well as the global increase in paternal age at conception, may also increase both the genetic, epigenetic, and mutational load carried by the offspring. Finally, in modern societies that have gone through the demographic transition, there is no longer intense selection pressure for high-fertility genes, with the result being that human fertility will inevitably decline. Furthermore, the increasing use of medically assisted reproduction preserves poor fertility genes in the population. This complex interplay of social, economic, cultural, and biological factors is serving to drive down global fertility rates in our species to below replacement levels. These trends will carry long-term implications for governments, national health service providers, and the populace at large. GDP, gross domestic product.

As a result of all these factors, fertility and potentially fecundity of our species will continue declining in the future. Figure 1 summarizes some of the factors discussed in this segment that have a high impact on MRH.

### What is happening around the world in MRH: a whistle stop tour

To assess what is happening in MRH around the world, an analysis was carried out of Africa, Australia, China, European Union (EU), North and South America. For comparative purposes, Supplementary Table S3 presents some demographics about these regions.

#### Africa

Africa is a vast and diverse continent comprising 54 recognized countries with an overall low concentration of wealth, and a high prevalence of male infertility owing to untreated sexually transmitted infections (STIs), unhealthy lifestyle choices, obesity, and environmental pollutants (Sarkodie, 2018; Frank *et al.*, 2023; Legese *et al.*, 2023). There is a strong argument to prevent infertility and to provide treatment as infertility is a medico-socio-cultural problem associated with gender-based suffering (Serour and Serour, 2021). While conventional methods for treatment of male infertility are available and accessible to varying degrees, medically assisted reproduction (MAR) technologies availability, accessibility, and affordability for moderate and severe male infertility treatments are deficient in many African countries (Serour *et al.*, 2019). Studies have reported a disproportionately high percentage of male factor infertility MAR cycles (58%), whether as a primary or secondary indication (Archary *et al.*, 2023). In 2019, the African Registry of Assisted Reproduction (ANARA) reported that 225 925 MAR cycles were performed in Africa. This equates to a significant underserving of medical need, based on calculations that 1500 MAR cycles/million population/year is a minimal requirement (assuming that only 50% of infertile couples would opt to have MAR; Fauser *et al.*, 2002); thus, the minimal needs for Africa is 1 980 000 cycles/year. The reasons for huge disparities between needs and access include high cost to establish and maintain MAR centers, a low number of trained African reproductive medicine experts, and a high cost for MAR cycles compared with gross domestic product (GDP). In addition, MAR technologies are not covered by health insurance, there are problems with importing disposable MAR equipment and high cost of drugs, and availability of MAR centers in the public sector is scarce, and mostly available in the private sector.

There are a number of opportunities to improve the current situation. Among them are health education and lifestyle improvement programs to prevent disease and thus reduce the need for MAR, one-stop clinics for infertility investigations, and the use of alternative treatments for mild and moderate male infertility, such as soft stimulation protocols and low-cost IVF laboratories (Ombelet *et al.*, 2022, 2023). However, many other challenges remain, such as persistent unhealthy lifestyle, environmental pollutants, socio-cultural, and religious barriers for some modalities of male infertility treatment, the high cost of MAR medications, scarcity of MAR centers in the public sector, lack of coverage of infertility treatment by health insurance policies, and lack of economic support by international agencies and philanthropies to support infertility treatment in Africa.

#### Australia

Australia is unique as the only nation with a nationally supported MRH program named Healthy Male (formerly Andrology Australia), a not-for-profit organization established in the year 2000 as a center for excellence in male health. Pleasingly, it is now seen as the ‘first port of call for information’ by the community, as well as by health and medical professionals and government.

Healthy Male was commissioned to lead the development of the National Men’s Health Strategy in 2018 because of its reputation for the provision of evidence-based information. The literature review and consultation process led to the release of ‘The Current State of Male Health in Australia*:* Informing the development of the National Male Health Strategy 2020–2030’ (Commonwealth of Australia (Department of Health), 2019) and identified specific actions to address health issues throughout men’s lives.

Healthy Male engages with many partners to achieve specific outcomes, for example with the Fertility Society of Australia and New Zealand to ensure evaluation of infertile men in MAR units, collection of national data on etiology, and research on behavioral and lifestyle influences in male factor MAR. In addition, Australian Men’s Shed Association operates more than 1000 local sites and provides quality health information. The Federal Government also supported ‘Plus Paternal: A focus on fathers’, a project aimed to acknowledge, engage, and support men as fathers and prospective fathers (Healthy Male, 2020).

#### China

In 2015, the one-child policy was expanded to a two-child policy owing to fears of a further decline in birth rates. However, birth rates continued to decrease to 12% in 2017 and further down to 7% in 2021 and reached a negative population growth in 2023 (https://data.stats.gov.cn/english/). Simultaneously, China is also facing a problem of a skewed sex ratio; the current population sex ratio is 104.3 (as per 1 July 2021; males per 100 female) compared to the UK (97.6) and the USA (98.2) (UN, Department of Economic and Social Affairs population division). To address the population problems, the State Council published the strategic plan for ‘Healthy China 2030’ (Chen *et al.*, 2019), with stated goals of equalizing the newborn sex ratio to achieve sex balance, applying family planning and social support, and developing a mature population monitoring mechanism by 2030. Because the Chinese Government is crafting pro-natalist policies, it is essential to study MRH in China. At least in part because of China’s clear goal to improve reproductive health, e.g. sex balance by 2030, the number of MAR cycles per year has increased from ∼200 000 in 2009 to 1.2 million in 2021 (Qiao *et al.*, 2021). The diagnosis of male factor infertility in 52% of couples underscores the importance of addressing MRH through enhanced research and education efforts. This need is further emphasized when considering the scale of the challenge; based on the estimation that two to three semen analyses should be performed prior to MAR treatment, 2.4–3.6 million semen analyses were performed in 2021. In 2022, andrology services became even more widely available to support 536 MAR clinics and 27 sperm banks, and for semen analysis in non-MAR diagnostic laboratories. On the benefits side, the large number of male factor cases requiring MAR will be generating an enormous clinical dataset for data mining, and a large amount of discarded biological material, as a potential source for laboratory research. Moreover, the Chinese Society for Reproductive Medicine (CSRM) is a national society for all reproductive medical specialists. It provides updated information and maintains standards by organizing annual conferences, training, and onsite external quality assurance (EQA) programs. While of great benefit, there is, however, no national EQA program for individual centers to join, and there are no central databases or andrology registries available. To ensure the quality of MAR services, China currently has produced well-defined guidelines for MAR settings such as the scale of service, personnel requirement and training, and number of centers per given population (775 cycle per million population on average, from 2018 data), but there is an unequal distribution of MAR facilities (Qiao *et al.*, 2021).

One aspect of China that is unique in comparison to the rest of the world is its investment in funding for MRH research. In fact, the national Natural Science Foundation of China (NSFC), the primary research funding agency, allocated 4.25% of its budget for MRH between 2016 and 2021 (Liao *et al.*, 2020), which eclipses many other countries, such as the USA and the UK (Gumerova *et al.*, 2023).

#### European Union

EU countries have some coordination regarding health and research topics (European Commission, Directorate-General for Communication, 2017), including offering fertility monitoring, surveillance and follow up as well as conducting coordinated research on MRH within a common framework, and funding opportunities in Europe (i.e. Growsperm). In recent years, there has been a growing interest in MRH among EU countries, as evidenced by patient support organizations such as Fertility Europe (FE) (see below). In addition, there is strong support for MRH by scientific societies led by ESHRE and the European Andrology Academy (EAA), and several EU initiatives focused on MRH, among them COST and Action ANDRONET. Despite this, European research funding for MRH remains low (Barratt *et al.*, 2021). More positively, the EU has a number of EQA schemes in andrology and a well-established register of MAR units. However, there are regulatory differences among countries, and a recent survey revealed that substantial differences exist between European regions in social knowledge/awareness concerning MRH (De Jonge *et al.*, 2023).

#### North America

Using the 2021 USA census data for people of reproductive ages 25–40 years and WHO recent values releases about infertility prevalence (WHO, 2023b), more than 10 million couples in the USA likely suffer from subfertility, among whom 3–4 million are men. Despite this demand, a number of barriers restrict or prevent access to reproductive health care (Mehta *et al.*, 2016). To address the need for more specialists, 15 andrology fellowship programs in the USA and one in British Columbia, Canada, are coordinating their efforts (information in American Society of Reproductive Medicine (ASRM) website).

In the USA and Canada, there is no national mandate for infertility coverage. In the Canadian provinces of Quebec and Ontario, public funding has been made available for at least the first cycle of MAR. In the USA, 22 states have some form of infertility and/or fertility preservation coverage and, of those, 15 states mandate coverage for IVF (information in Resolve website). Specifically, patients in the USA and Canada suffer because of inequity in insurance coverage by most states, and male infertility is often excluded from health insurance policies (Dupree *et al.*, 2016). Of concern is that without health insurance coverage for male infertility, there are missed opportunities for the diagnosis of associated ill health conditions (please refer to ‘Reproductive and somatic health’ section).

The USA has a national IVF registry coordinated by the Centers for Disease Control and Prevention (CDC). In 2021, a total of 413 776 MAR cycles (∼238 126 patients) were reported by 453 clinics; 80% of total patients were ≤40 years of age, and 27.8% of total MAR cycles were attributed to a male factor diagnosis. Surprisingly, 80% of these cycles were ICSI, suggesting that ICSI may be used out of fear of an occult male factor for which diagnostic tests do not exist. This brings into question the status of basic and translational research funding for male infertility in the USA. Per capita spending on MRH research in the USA ranks high compared with other countries (De Jonge *et al.*, 2022). When fiscal years 2016–2019 of the USA National Institute of Child Health and Human Development (NICHD) were evaluated, only 2.6% went toward funding infertility research (Gumerova *et al.*, 2023). This funding level seems remarkably low given that: infertility is classified as a disease but ranks much lower than other disease states in federally supported funding; infertility occurs in relatively high frequency and can be considered a public health issue; and infertility may be a precursor to other health-related illnesses (see ‘Reproductive and somatic health’ section).

The development of new contraceptive methods has been slow in recent decades, with the burden largely falling to the woman. Recently, the US Supreme Court’s ruling on the right of a woman to seek abortion has resulted in an increase in the number of young men seeking vasectomy, indicating that men want to be involved in family planning (Bole *et al.*, 2023). The US market research demonstrates that men and women have significant interest in and comparable desire for non-hormonal, reversible male contraceptives (see ‘New methods of male-based contraception’ section).

The USA Government provides funding and support for the Office on Women’s Health (OWH), which coordinates women’s health initiatives across the Department of Health and Human Services. To demonstrate and prioritize the social and economic importance of MRH, legislation should be passed to establish an Office on Men’s Health (OMH) with the vision of: ‘all men and boys achieve the best possible health’ (Miner *et al.*, 2018). For men’s overall and reproductive health care seeking and needs to be met, there must be an investment by the government to formulate policy changes and provide funding to support reorientation of men’s health care services (Shand and Marcell, 2021), and that starts with identifying how men best communicate.

#### South America

Although there are no MRH regional policies in South America, there are well-established organizations, such as the Red Latinoamericana de Reproducción Asistida (REDLARA), that provide continuing education to all member institutions. The Latin American Registry of Assisted Reproduction (RLA) is part of REDLARA and publishes the results of MAR procedures from certified centers. The 2019 RLA reported a total of 54 797 MAR cycles among their centers, of which 26.9% (n = 14 747) were male factor infertility cases, highlighting the relevance of MRH in the region. RLA is part of the International Committee for Monitoring Assisted Reproductive Technologies (ICMART), and participates in a program of South–South cooperation with ANARA, to benefit infertility institutions and people in both continents (Chambers *et al.*, 2021). South America also has several patient organizations, among them CONCEBIR, which recently established a program called CONCEBIR ‘Cosas de Hombres’ (CONCEBIR ‘Men’s Stuff’). In addition, Red TRAscender is a Latin American network that raises awareness about infertility and connects patients with health professionals and government agencies. Some countries (i.e. Argentina, Brazil) have developed legislation for the provision of public access to MAR and/or male contraception-related procedures (i.e. vasectomy and semen cryopreservation prior to surgery), although with striking policy differences among them that impact a person’s reproductive options.

A challenge for the region is the impact of unequal social and economic development across countries (Gavin *et al.*, 1998; Amarante *et al.*, 2016). Also, challenging male attitudes are still present, characterized by an hegemonic masculinity in male sexual and reproductive attitudes and relationships (Díaz-Rojas *et al.*, 2020) that may translate to low vasectomy rates (Sanchez-Molano *et al.*, 2019) and to a perception of no risk of STIs, highlighting the need for public health policies for prevention, early diagnosis, and treatment of STIs in young people (Vallejo-Ortega *et al.*, 2022). Additionally, there is a lack of andrology registries and regional EQA programs, and professional development relies on personal self-improvement. South America is also experiencing a trend toward a semen quality decline (Siqueira *et al.*, 2020; Rosa-Villagrán *et al.*, 2021; Levine *et al.*, 2023), and the impact of age, obesity, lifestyle, and environmental factors upon semen quality (Verón *et al.*, 2018) is a regional threat, as reported in other regions. Nevertheless, scientists and health professionals are interested in integrating into large networks to improve MRH. As an example, the network AndroLATAM was created in 2022 and associated with RedLARA, to strengthen communication between scientists, clinicians, patient groups, and policy makers. The Pan American Health Organization (PAHO) has been committed to support MRH programs (PAHO, 2020); it recently participated in preparing the Spanish translation of the WHO manual for semen evaluation 6th Edition, and is planning in the future to contribute to professional training in MRH.

While there are substantial differences in MRH among global regions, understanding these differences is crucial for implementing targeted interventions and strategies to improve MRH worldwide. Collaborative efforts between governments, healthcare organizations, non-governmental organizations, and communities are required to address the specific challenges and promote better reproductive health outcomes for men globally.

## MRH: science and medicine

### The sperm epigenome—considerations for the next generation and beyond

Male infertility is intertwined with environmental exposures, including toxicants, and lifestyle factors such as being overweight, cannabis use, alcohol, and poor diet (Barazani *et al.*, 2014; Lismer and Kimmins, 2023). Epidemiological studies suggest that the paternal environment may impact the health of future offspring (Pembrey *et al.*, 2006; Kaati *et al.*, 2007). The sperm epigenome has since been identified as a connecting molecular link between environmental factors, male infertility, and offspring health (Fitz-James and Cavalli, 2022; Lismer and Kimmins, 2023).

During spermatogenesis, specialized epigenetic mechanisms serve crucial roles in multiple aspects of germ cell development and are critical to maintain the gamete genetic and epigenetic quality (Kimmins and Sassone-Corsi, 2005). The disruption of epigenetic pathways can impact fertility, embryonic gene expression, and affect the health of subsequent generations (Siklenka *et al.*, 2015; Ly *et al.*, 2017; Lismer *et al.*, 2021).

The state of knowledge regarding the role of the human sperm epigenome is in its infancy as obtaining precious reproductive cells, embryos, and clinical data is difficult. Nonetheless, there is a high degree of conservation in the sperm epigenome between mice and men and indicators that transmission of a molecular memory of exposures may occur by similar mechanisms of inheritance (Erkek *et al.*, 2013; Lambrot *et al.*, 2021). Identifying the mechanisms linking parental exposure to offspring development and health has enormous potential for novel disease prevention routes.

### Enhancing genetic studies for infertility: personalized medicine in MRH

Understanding the underlying cause of a disease is essential in order to tailor treatment and to predict and prevent co-morbidities. Unfortunately, for the majority of infertile men, the underlying molecular cause is unknown. Therefore, the success of MAR cannot be predicted, the transmission of infertility through MAR cannot be predicted, and the association between infertility and increased morbidity and mortality cannot be unraveled.

When it comes to diagnosing genetic causes of male infertility, the field is in the initial stages of reaping benefits from the genomics revolution. However, some practice guidelines still refer to outdated genetic procedures such as karyotyping and Sanger sequencing of individual genes. This is in contrast to other fields in medicine, including rare diseases and cancer, where genome sequencing has become a first-tier test. Clearly, this also offers opportunities for MRH, because as genomics has become more affordable and analysis more automated, the ability to diagnose a medical condition, such as male infertility, has increased. Investment and international collaboration are essential to support this endeavor, such as the International Male Infertility Genomics Consortium (IMIGC). Through this kind of international collaboration, including active data sharing and combining expertise from basic discovery to clinical diagnostics, an increasing number of validated genetic causes of male infertility has been reported (Oud *et al.*, 2019, 2022; Houston *et al.*, 2021). While progress is very promising, many infertility sub-types are yet to be analyzed and knowledge gaps remain.

Equally for the identification of some types of male infertility, notably dominant genetic causes, it is essential that patient–parent genome sequencing is incorporated into the diagnostic pipeline. For all types of male infertility, the sharing of sequence data across the global research community is essential. If done correctly, genomics research, diagnostics, and therapy development will go hand in hand in order to make the progress that will allow specialists to properly diagnose a large fraction of infertile men and provide useful information to these men and their partners. Uncovering the genetic origins of male infertility holds the promise of paving the way for drug development, with the potential to either enhance fertility or inhibit it, thus contributing to advancements in both fertility treatments and contraceptive development.

### Reproductive and somatic health

Major medical organizations worldwide recommend that it is essential for both members in an infertile couple be evaluated to obtain a realistic understanding of causality (Schlegel *et al.*, 2021a,b). Despite this, a male infertility examination in the USA is bypassed up to 25% of the time and some couples are treated with MAR without the man having received an evaluation (Eisenberg *et al.*, 2013). Considering the increase in the use of MAR around the globe, with rates of up to 10% of children being conceived via MAR (European IVF Monitoring Consortium *et al.*, 2022), it becomes clinically and financially essential for a male infertility specialist to evaluate the male partner.

Moreover, while male infertility is a critical problem for couples facing difficulties conceiving, its public health relevance extends beyond fertility and reproduction. Male infertility may provide a window into later health and, as such, may be a harbinger of significant future medical problems. Indeed, current health and reproductive fitness are closely associated but reproductive health also seems to be a biomarker of future health; severe cardiometabolic conditions, genetic syndromes, or cancers appear to be increased in men with semen abnormalities (Eisenberg *et al.*, 2015a,b; Behboudi-Gandevani *et al.*, 2021; Hansen *et al.*, 2023). There are, however, several important knowledge gaps with this emerging literature. First, the excess premature mortality observed among infertile men cannot be explained by the excess risk of cardiometabolic conditions or testicular cancer alone, suggesting that male factor infertility may also be related to a higher risk of other conditions that directly or indirectly result in early death. Second, the majority of the literature has only evaluated crude proxies of male reproductive function, such as fatherhood, infertility diagnosis or type of fertility treatment, with only a few studies evaluating direct markers of testicular function. Third, markers of Leydig cell function together with semen quality have been understudied in relation to later life health outcomes. Given these knowledge gaps, it is presently unclear whether the associations between semen quality and increased morbidity are specific to spermatogenesis or reflective of a broader phenotype of impaired testicular function. Furthermore, there is an important overlap between predictors of semen quality and risk factors for cardiometabolic disease and premature mortality including obesity, smoking, physical inactivity, and poor diet (Latif *et al.*, 2018; Batty *et al.*, 2019). Therefore, it is critically important to identify the extent to which the relation between testicular function and long-term health in men is explained by shared lifestyle and biological factors, respectively. As we examine ways to incorporate such findings into clinical practice, the etiology of the association must be identified to create a program to hopefully mitigate the increased health risk that infertile men face. In that regard, supporting evidence for a genetic link between spermatogenic maturation arrest and cancer predisposition was reported in a multicentric study (Krausz *et al.*, 2020).

Answering these critical questions will advance knowledge and generate actionable information to guide the treatment of infertile men, and men with impaired testicular function, beyond immediate reproductive needs.

### New methods of male-based contraception

**Figure 2.**
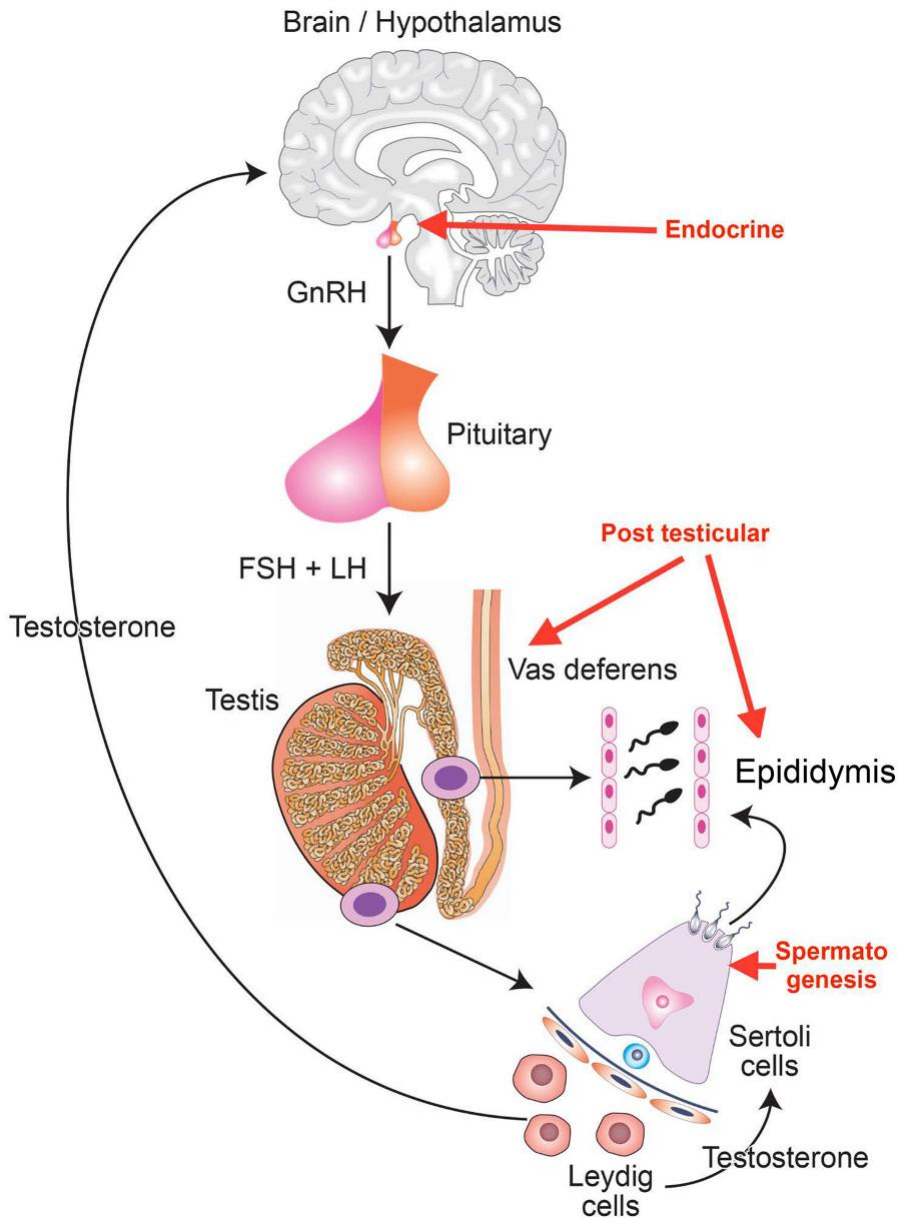
**Targets of contraception in the male reproductive tract.** Schematic of the male reproductive system showing the main targets areas for novel contraceptive approaches (red text and arrows), specifically the endocrine approach resulting in suppression of GnRH and LH/FSH, approaches targeting spermatogenesis, and post-testicular approaches targeting the epididymis and vas deferens. Adapted from Reynolds-Wright JJ, Anderson R. *BMJ Sex Reprod Health*. 2019; 45: 236–242.

The male reproductive system offers a multitude of potential targets for contraceptive development, conveniently divided into those affecting endocrine control of the testes, those primarily targeting spermatogenesis, and post-testicular approaches, i.e. sperm function (Fig. 2). The hormonal approach is by far the most developed and is now in advanced clinical trials, whereas other approaches remain generally at pre-clinical investigation (Nya-Ngatchou and Amory, 2013; Reynolds-Wright and Anderson, 2019). Surveys consistently demonstrate men’s interest in new approaches to fertility regulation and their willingness to use them, and while there is a limited literature on women’s attitudes, it strongly supports that they would value greater availability of male methods (Reynolds-Wright *et al.*, 2021). This is supported by current experience in recruiting couples to a contraceptive efficacy study where the most common reason cited for taking part is that men should be able to contribute more to contraceptive practice within a relationship.

Hormonal approaches based on suppression of gonadotrophin secretion have been investigated for over half a century (Reynolds-Wright and Anderson, 2019). The ability of testosterone to reversibly suppress spermatogenesis was developed into landmark contraceptive efficacy studies by the WHO, demonstrating that testosterone administration resulted in azoospermia in the majority of men, and that this then allowed excellent contraception for a 12-month period (WHO, 1990).

The arrival of new testosterone preparations, particularly long-acting testosterone undecanoate (TU), provided a great spur to development. Organon and Schering jointly conducted studies using TU with specially formulated etonogestrel implants (Mommers *et al.*, 2008) and the WHO with the organization named Contraception, Research and Development (CONRAD) were developing the combination of TU with norethisterone enanthate (Behre *et al.*, 2016). Currently, an efficacy trial of an alternative approach of daily self-administered gel containing testosterone with Nestorone (segesterone) is underway, run by the NICHD and the Population Council. This method induces dose-dependent suppression of spermatogenesis. Preliminary reports from this international study should be presented in 2024.

A further approach might improve on vasectomy. The RISUG (reversible inhibition of sperm under guidance) method involves injection of styrene maleic anhydride into the lumen of the vas; this is not occlusive but disrupts the spermatozoa as they pass through. It is also potentially reversible by injection of the solvent. Initial data suggest high efficacy, but no further publications on this have emerged recently. A similar approach, Vasalgel, involves injection of styrene alt-maleic acid, which unlike RISUG is occlusive yet also potentially reversible by injection of the solvent. This has been demonstrated in animal models, and in 16 treated rhesus monkeys no conceptions were observed in over 1 year of exposure (Colagross-Schouten *et al.*, 2017). A key aspect of this approach is the need for accurate injection into the vas, with leakage resulting in local inflammation. This method is currently under development for clinical trials, supported by the Male Contraceptive Initiative.

Testicular and post-testicular approaches target a wide range of pathways including retinoic acid synthesis and action, chromatin remodeling through bromodomain acting drugs, interfering with spermiation through lonidamine derivatives, sperm maturation in the epididymis, and sperm protein targets related to male gamete function(s) (Nya-Ngatchou and Amory, 2013; Balbach *et al.*, 2023; Mariani *et al.*, 2023; Nickels and Yan, 2023). An early clinical trial of an orally active retinoic acid receptor alpha antagonist YCT-529 has recently been initiated by Your Choice Therapeutics, providing the possibility of a large step forward toward a non-hormonal male contraceptive.

Despite this wealth of knowledge and progress over many years, it remains the case that a novel male contraceptive remains unavailable. The cited WHO studies confirm the potential of hormonal male methods to provide highly effective contraception. There remains limited commercial investment in both this approach and in contraceptive development in general: near perfect efficacy is required with near zero side effects, unlike other medical products. Participation by both men and their partners in trials, however, confirms that there is market demand. There is also the novel regulatory aspect of one person taking a drug to prevent a condition (i.e. pregnancy) in another, needing development of the concept of shared risk. That not all men show sufficient suppression of spermatogenesis in the hormonal approach remains an issue, and that time for suppression is required. Nevertheless, there is optimism that existing approaches, notably the testosterone/Nestorone gel, will advance to extensive Phase III clinical studies for registration, ultimately paving the way for widespread availability.

## MRH: society, economics, policy, and education

### Common threats to MRH

Narratives in popular culture, e.g. newspapers, broadcast media or in social media, depict many threats to MRH. The topics typically resonate with common lifestyle factors (e.g. increasing age of paternity, smoking, consumption of alcohol and recreational drugs, urogenital infections, occupational risks) and impact directly on sperm quality and have been well reviewed in the literature (Gimenes *et al.*, 2014; Ricci *et al.*, 2017; Jimbo *et al.*, 2022). However, there are other relevant threats, some of which are described below.

#### Potential decline in semen quality

There are data that suggest that sperm quality (typically sperm concentration) has declined in recent decades (Levine *et al.*, 2023), although this is the subject of considerable debate (Jørgensen *et al.*, 2021; Auger *et al.*, 2022; Cipriani *et al.*, 2023). If true, then a decline in sperm concentration, or quality, in future fathers increases the likelihood of infertility, given the known relation between sperm concentration and probability of conception (Bonde *et al.*, 1998; Keihani *et al.*, 2021). This is likely to become increasingly profound when combined with increased male age before pregnancy is attempted, as outlined above.

#### Less sex in young males

Regular (unprotected) intercourse is a critical part of achieving a natural pregnancy, yet there is strong evidence that young men and women are having less sex than their parents’ generation that puts them at risk of infertility worldwide (UK: Mercer *et al.*, 2013; Australia: de Visser *et al.*, 2014; Finland: Kontula, 2015; Japan: Japan Family Planning Association, 2017; USA: Ueda *et al.*, 2020). For example, the UK’s National Survey of Sexual Attitudes and Lifestyles shows that, over the past two decades, there has been a decrease in how often people (aged 16 to 44 years) say they have sex (Mercer *et al.*, 2013). The same trend was reported by the General Social Survey of young people in the USA, and is largely being driven by a decreased incidence of sexual intercourse by young males (Ueda *et al.*, 2020). Regarding the explanation(s) for such change in attitudes toward sex in young men, Twenge (2020) analyzed several potential factors and proposes two primary explanations: first, adolescents and young adults are taking a longer time to transition into adulthood and, second, the growth of the internet and digital media since 2000. Acknowledging the challenges associated with conducting randomized clinical trials in cultural change research, determining definitive explanations for the observed decline in sexual activity becomes inherently complex. However, if this trend is replicated in other countries or intensifies further, it could potentially lead to more pronounced declines in birth rates.

#### Poor data collection

Studies investigating threats to MRH are few and far between, they provide contradictory results or are poorly designed to drawn robust conclusions, as recently brought into sharp focus by an analysis of the 100 largest randomized controlled trials in male infertility published between 2010 and 2021 (Rimmer *et al.*, 2022). This has been reinforced by Björndahl *et al.* (2022), who have published a plea for higher standards in the design and publication of data from semen analysis.

### Public information and interaction with patients

Male engagement with health services is thought to be quite poor, particularly in the arena of MRH, where most men have inadequate knowledge about the limitations of female and male fertility, and overestimate the chance of natural and assisted conception (Hammarberg *et al.*, 2017). For many men, fertility issues are seen as a private matter (see below) and, for that reason, it has been proposed that the internet and social media may provide a suitable route to obtain information about their reproductive health. In a study of young people (aged 13–18 years), Goodyear *et al.* (2022) found that there were two main purposes of engagement with social media: communicating with friends; and accessing health-related information. Moreover, irony and humor were central learning mechanisms, and acceptable ‘banter’ allowed young men to engage with health discourses without fear of peer ridicule. Social media now dominates our lives and data from Global Web Index (accessed 8 February 2022 at 15:12 GMT) shows that young people aged 16 to 24 years now spend over 3 hours per day using it. While this decreases with increasing age, those aged 45 to 54 years still spend 1.39 h/day on social media.

The advantage of social media for professional groups to communicate with the public and patients about MRH is that scientists, doctors, and nurses are generally regarded to be honest and have integrity (Clemence and King, 2022), and are therefore trusted by the public. This is a concept recently reinforced by the Wellcome Trust Global Monitor Report 2020 (Craig *et al.*, 2021). Studies such as Daumler *et al.* (2016) found that more than 50% of men expressed an interest in obtaining more information about MRH, citing medical professionals and online sources as their preferred sources, providing a green flag for professionals to communicate to the public and patients via social media.

### MRHI global questionnaire

**Figure 3.**
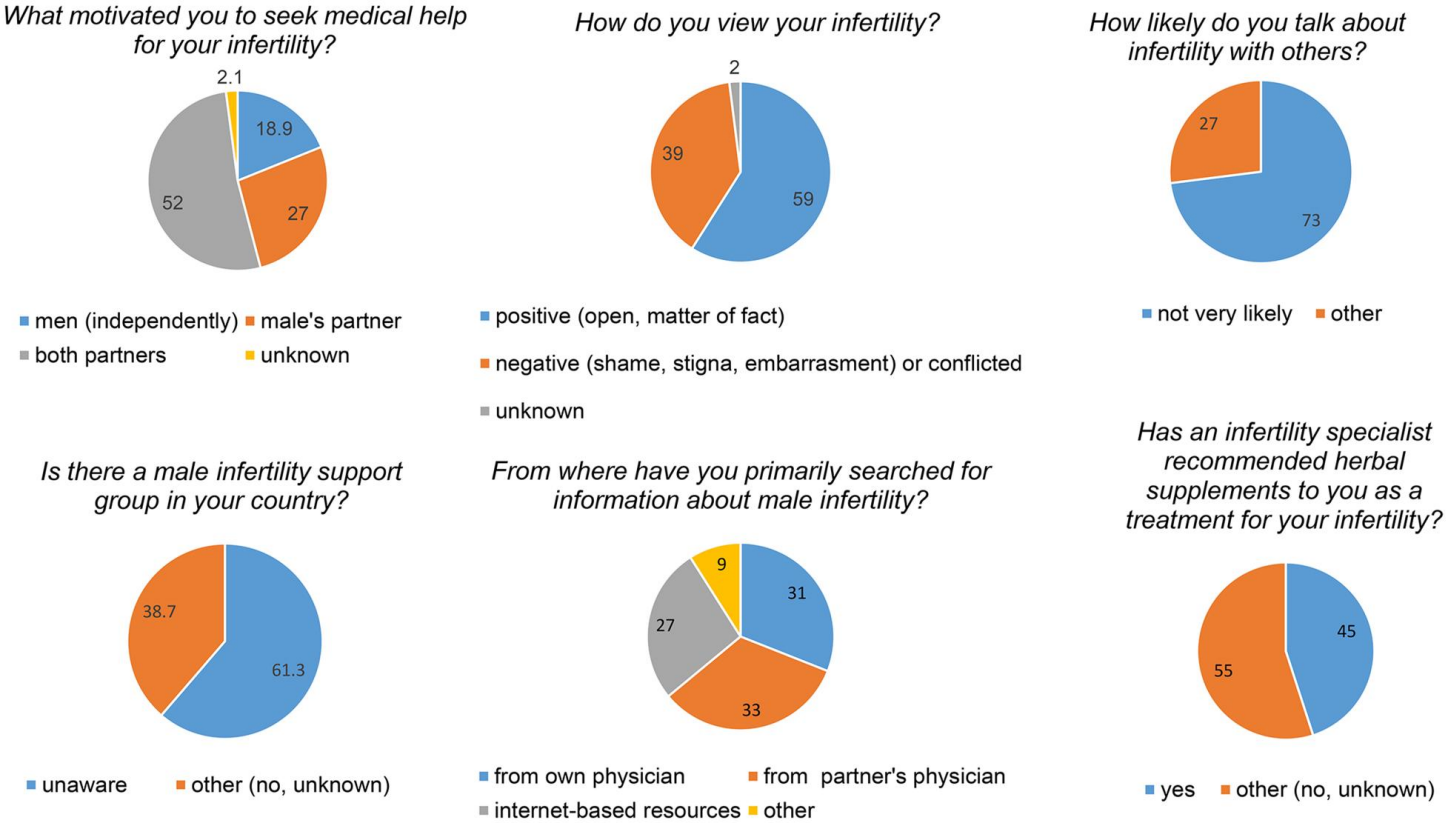
**Results of the Male Reproductive Health Initiative questionnaire about male infertility.** In a 1-year period, 1171 men aged 20–59 years responded to the questionnaire. Collectively, men 30–39 (60.8%) and 40–49 (23.9%) years of age made up 85% of the age demographic. Patients responded to: how they feel about their infertility; what motivated them to seek health care; how likely are they to talk with others about their infertility; their awareness of male infertility support groups; what their primary source for information is regarding male infertility; and has an infertility specialist recommended herbal supplements to you as a treatment for your infertility? The questionnaire remains open to collect data and can be accessed using the following link: https://fertilityeurope.eu/male-infertility-questionnaire-participate-now/.

As previously mentioned, in addition to reproductive health concerns (De Jonge and Barratt, 2019), there are psychosocial knowledge gaps that require investigation, and that is: what are men’s attitudes regarding their infertility? Recently, the MRHI conducted a University IRB-approved study to ask infertile men from around the world: how they feel about their infertility; what motivated them to seek health care; how likely are they to talk with others about their infertility; what is their awareness of male infertility support groups; what their primary source for information is regarding male infertility; and has an infertility specialist recommended herbal supplements to you as a treatment for your infertility? These questions were presented in the form of an anonymous questionnaire translated into 20 European and Asian languages. A summary of the findings is presented in Fig. 3. These results have provided greater clarity about men’s diverse feelings about their infertility (De Jonge *et al.*, 2023). The study emphasizes the importance of men better identifying their emotions and more openly sharing their feelings, especially with their partner and with other men. Addressing their feelings, receiving support, and gaining knowledge about their infertility will help men to feel emotionally stronger, empowered, and have a more positive state of mind, and they might be in a better supportive position for their partner. The information can also be used by reproductive and mental health practitioners to support men in being more engaged individually and collaboratively with their partner throughout the MAR journey.

### Developments in men’s health policy

Men’s health is unnecessarily poor across the world. At the global level, male life expectancy is 5 years lower than female life expectancy (WHO, 2023a). Globally, the risk of a 30-year-old person dying from any of the four major noncommunicable diseases—cancer, cardiovascular disease, diabetes, and respiratory diseases—before reaching the age of 70 years is 22% for men and 15% for women (WHO, 2019). For cancer alone, the all-cancer incidence rate is 18% higher for males and the mortality rate 31% higher (Sung *et al.*, 2021). The male suicide rate is twice as high as the female rate globally (Ilic and Ilic, 2022) and for every 10 female deaths from coronavirus disease 2019, there have been 13 male deaths (Global Health 50:50, 2021). Healthier men would significantly reduce costs and workloads for currently overstretched health services. Better men’s health would also be good for women and children and the wider world of local communities and workplaces.

Despite the potentially huge social and economic benefits, as well as the ethical case for action, men’s health has not been systematically addressed by health organizations at all levels (Baker, 2020; Baker *et al.*, 2020). In part, this is because health policymakers and practitioners have not been trained to take account of sex and gender, and they are therefore not seen as significant issues for most global and national health organizations. There is also resistance to addressing men’s health because men are held to be responsible for their own health problems even though it is not easy for an individual man to step outside of the ‘gender rules’ he has been expected to follow since early childhood. The better news is that there are now some clear opportunities for progress in men’s health. Growing media coverage and initiatives like Movember and Men’s Health Week have given the issue a much higher public profile. Men’s health is no longer seen as just being about sexual and reproductive health; rather, it is about any aspect of men’s health impacted by sex and/or gender. This includes mental health, where men’s difficulties with disclosing their concerns to family and friends as well as health professionals are very clear and now widely understood. The WHO, both at the global and regional levels, is now showing more interest in men’s health (see ‘Sustainable development goals and MRH’ section). The WHO published a men’s health strategy in 2018 (World Health Organization, 2018). Seven countries—with Ireland being the first in 2009—have published national men’s health policies; there is evidence of their positive impact (Baker, 2015), and they provide a platform for similar initiatives elsewhere. There is also a growing body of academic research, much of it published in specialist men’s health journals, which has demonstrated the effectiveness of a wide range of practical interventions.

Furthermore, Global Action on Men’s Health (GAMH) is an international non-governmental organization aimed to elevate men’s health on national and global health policy agendas. There is an opportunity for GAMH and international societies, such as ESHRE, to raise long-neglected issues concerning male fertility and to bring about improvements in male health that will also have important spin-off benefits for reproductive health. By implementing male-responsive strategies and engaging in dialogue and collaboration with men’s health advocates, organizations and researchers, the policymakers can help to accelerate improvements in men’s health and work toward improving the overall health and well-being of men in their communities around the world.

### The broader economic consequences of infertility

The consequences of being childless, or unable to achieve one’s desired family size, can not only impact couples but also, when viewed in aggregate, i.e. at population level, can pose economic consequences for households, the government, and the broader economy. By understanding how all society members are linked together through economic institutions and transactions, especially as it relates to many public benefits programs that are funded through pay-as-you-go taxes, we gain better insights into the broader effects that a failure to balance family size can have on others.

The present demographic transition from high to low fertility and increasing longevity places excess demand on the remaining workers to fund public programs that can create fiscal drag for the economy at large. Specifically, it is the younger aged cohorts that carry the fiscal burden of older generations. It is important to recognize that all government promises now, and into the future, must be paid for by current and future workers. When the balance of workers (those paying tax) is overwhelmed by those who do not work (pay tax), economic problems ensue. Notably, in the face of declining global birth rates, children conceived by MAR augment the population of naturally conceived children and contribute to the cost of caring for the ‘non-employed’ members of society. All things being equal, when more children are born in society, the need for higher taxes is reduced, thereby reducing the lifetime net taxes paid by individuals (Rizza and Tommasino, 2010).

Through this lens, we can see how MAR-conceived children, although a small contribution to overall national births, can help reduce the likely tax liabilities paid by naturally conceived children. This would suggest that infertility is not only a problem faced by couples. In fact infertility, if successfully treated, has economic consequences that benefit society as a whole. Furthermore, as children are positive net tax contributors to government over their lifetime, public investments in MAR that enable people to achieve their desired family size represent positive net fiscal value from which all society members will benefit (Connolly *et al.*, 2008; Lee and Mason, 2011).

To compensate for the effects of ageing populations, governments often turn to pronatalist policies to influence birth rates by reducing the financial burden of raising children (Sobotka *et al.*, 2019). The effects of pronatalist policies achieve varied results, and are often considered expensive to implement. A recent review of pronatalist policies offering child benefits equal to 10% of household income increased birth rates from 0.5% to 4.1% (Stone, 2020). By comparison, annual reporting of the contribution of MAR-conceived births is equivalent at the least, and is likely to be greater than most pronatalist policies in those countries with well-funded programs for treating infertile couples (European IVF Monitoring Consortium *et al.*, 2022). Considering the contribution of MAR-conceived births to national births, several countries have changed the funding of MAR for the sole purpose of increasing birth rates. This would suggest that policy makers seeking to influence birth rates may be wise to consider a broader range of policies for influencing national births.

### Policy makers and how to influence change

Navigating infertility poses significant emotional challenges. Patient support organizations serve as invaluable resources, facilitating the sharing of experiences, feelings, and concerns among individuals and couples facing similar struggles. Patient support organizations actively contribute to pursuit by patients of equal access to fair and safe treatment. By fostering a sense of community, these organizations help alleviate feelings of isolation and offer crucial emotional support (McNaughton-Cassill *et al.*, 2000; Malina *et al.*, 2019; Soleimani *et al.*, 2023). Globally, numerous national infertility associations exist, such as FE, RESOLVE, Asociación Red Nacional de Infértiles, TRAscender, CONCEBIR, and more. FE stands out as an exemplary representative organization, bringing together 31 European national infertility member associations from 28 countries. This organization successfully unites diverse demographics, working collaboratively toward the common goal of patient advocacy.

FE aims to improve the rights of those affected by infertility, build a strong cross border network among European patients and member associations, share best practices, promote social change regarding the perception of infertility, and promote education in the field of reproductive health care. FE also aims to promote patient empowerment, the fight against health inequalities and discrimination, the support of quality care, patient safety, and patient-centered treatments, as well as the development of ethical guidelines and regulations within each European country. FE estimates that in the EU alone there are about 25 million people of reproductive age with infertility (FE website URL, Supplementary Table S1).

FE has been very proactive in engaging with stakeholders and policy makers on a regional national level to ensure that patients’ voices are heard. In 2020, FE started a project to formulate a map that would cover all European countries and their fertility treatment policies. In November 2022, a European Atlas of fertility treatment policies was published (European Atlas for Fertility Treatment, 2021), which shows clearly that regulations and funding of fertility treatments in 43 European countries are very diverse and often based on non-medical principles. One of FE’s biggest projects is the fertility awareness project that started in July 2021, aimed to prepare a web-based game for teenagers aged between 15 and 18 years and raise the players awareness of fertility but at the same time gather data for FE about the level of their knowledge. With the collected data available to our member associations, it can be used to advocate the importance of fertility awareness. Networking among infertility associations in Europe, and indeed globally, is essential. FE is also taking into account parties that are related to fertility and infertility, such as women's and men’s health organizations, ESHRE and other reproductive health professionals associations, educator organizations, and other patient organizations representing diseases related to infertility. Many working groups seek the perspective of patients, and invite FE as the patients’ voice.

### Training of the current and next generation in andrology

While andrology is increasingly recognized as the male counterpart to gynecology for the female, formal specialization in this discipline is still lacking in many countries. At the European level, the EAA is the main organization dealing with the promotion of education in clinical andrology, and has established 29 EAA-accredited training centers (also outside of the EU; URL list in Supplementary Table S1). Trainees can obtain the ‘EAA Clinical Andrologist’ title after a period of 18 months training in one of the accredited centers and an extensive exit exam. In addition, a number of permanent EAA Schools that provide hands-on training in ultrasound, basic clinical andrology, testis histology and pathology, and microsurgery for clinicians are available (https://www.andrologyacademy.net/education). In the USA and Canada, male fertility is taught as a structured fellowship after a urological residency. The Society for the Study of Male Reproduction within the American Urological Association is charged with matching applicants to programs and ensuring the educational quality of the fellowship programs. Many enter academic practice in urology residency training programs so that the majority of urology residencies have as a component male reproductive medicine. Promisingly, a network of 15 medical schools offering subspeciality training in andrology has been recently established to fill the void that exists in North America (see ‘What is happening around the world in MRH: a whistle stop tour’ section, North America). In the EU and globally, however, the number of male reproductive fertility specialists still lags well-being that of female fertility specialists (Najari, 2019).

There are at least three distinct areas for improving reproductive medical education. The first is in recognizing similarities and equal priority between male and female reproductive function and dysfunction, and in teaching both to all learners. The second is in multidisciplinary innovation; education in science has been an essential element of medicine for centuries, and reproductive medicine is no exception. And the third area for training is one generally not provided in medical education, but it represents a real need in reproductive medicine and in the world at large: we live in a world where politics and reproductive rights have become intertwined. It is essential that future generations of medical doctors are educated on how to succeed politically in the protection of medical care for all people.

Training and education need inspirational mentors. Dr Donna Vogel is a prominent leader in our field with an interest in male infertility and reproduction as well as basic and clinical research, and has been directing grant programs for 30 years. Dr Vogel has provided inspiration to a large number of people in the field at all levels of experience, and was asked to contribute a personal message about mentoring and leadership development, particularly to encourage and stimulate higher education students, young scientists, and clinicians. Supplementary Data File S3 presents her valuable reflections and guidance.

## What are the conclusions for the way forward?

One of the more challenging aspects of male infertility and reproductive ill-health broadly is its lack of visibility. The reasons for this are complex and involve deeply held cultural norms. Regardless, if governments and the public are unaware of the size of a problem, they are unable to prioritize it. Equally, the lack of visibility and knowledge may lead clinicians and pharmaceutical companies to overlook andrological disorders and fail to recognize the inherent benefit in treating such problems for individual men and society as a whole.

As academic leaders in andrology, the MRHI mission is to help clarify the types and scale of challenges associated with male reproductive ill-health, to define areas for urgent research focus and the types of resources, and ways of intelligence gathering required to answer such questions. To achieve this, during the course of 2021–2022, a working group was convened virtually, and key questions were formulated. Lead authors for each question were assigned and asked to assemble the most appropriate co-authors, including emerging leaders, to briefly summarize the current state of knowledge, to identify resource gaps and opportunities, and to develop a roadmap to improve MRH. The recommendations of this roadmap are deliberately high level and do not delve into specific fundamental research questions. It was accepted by all authors, however, that the attainment of a greater understanding of the mechanisms underpinning male fertility, spanning all relevant organ systems, is critically important to optimize male health and to manipulate male fertility in a targeted manner. Following review, the manuscript was published in *Nature Urology Reviews* under the title of ‘Frequency, morbidity and equity—the case for increased research on male fertility’ (Kimmins *et al.*, 2024). As outlined in the manuscript, Kimmins *et al.* (2024) presented a total of 13 urgent questions and 10 recommendations. Key topics include:

- Being able to precisely diagnose the etiology of male infertility and its frequency in a population, spanning all causes whether they be genetic or environmental.
- The appropriate use and limitations of existing therapeutic options and the formulation of additional targeted treatment options.
- Defining the whole of life health burden and economic cost of male infertility.
- Defining the consequences of MAR on child health across the lifespan.
- The development of male-based contraceptive strategies; and very importantly
- Improved education and communication strategies aimed at each of the public (across the lifespan), couples attempting conception, medical professionals, governments, and funding agencies.

Readers seeking comprehensive details are encouraged to consult the full paper.

Addressing these topics is an essential requirement for all andrology specialists, by fostering international cooperation and by facilitating the sharing of data and samples. If successful, the roadmap implementation holds the promise of advancing men’s health, offspring health, addressing imbalances in the treatment of male infertility—particularly where the female partner shoulders a disproportionate burden—and, over time, enhancing the efficiency of healthcare systems.

## Supplementary Material

hoae017_Supplementary_Data_File_S1

hoae017_Supplementary_Data_File_S2

hoae017_Supplementary_Data_File_S3

hoae017_Supplementary_Table_S1

hoae017_Supplementary_Table_S2

hoae017_Supplementary_Table_S3

## Data Availability

The data underlying this article are available in the article and in its online Supplementary Material.

## References

[hoae017-B1] Aitken RJ. The Infertility Trap: Why Life Choices Impact Your Fertility and Why We Must Act Now. Cambridge: Cambridge University Press, 2022a.

[hoae017-B3] Aitken RJ. The changing tide of human fertility. Hum Reprod2022b;37:629–638. 35079808 10.1093/humrep/deac011 PMC8977063

[hoae017-B4] Amarante V , Galván M, Mancero X. Inequality in Latin America: a global measurement. CEPAL Rev 2016;2016:25–44.

[hoae017-B5] Archary P , Potgieter L, Elgindy E, Adageba RK, Mboloko J, Iketubosin F, Serour G, Dyer S; African Network and Registry for Assisted Reproductive Technology. Assisted reproductive technologies in Africa: the African Network and Registry for ART, 2018 and 2019. Reprod Biomed Online 2023;46:835–845. 36959069 10.1016/j.rbmo.2023.01.014

[hoae017-B6] Auger J , Eustache F, Chevrier C, Jégou B. Spatiotemporal trends in human semen quality. Nat Rev Urol 2022;19:597–626. 35978007 10.1038/s41585-022-00626-w PMC9383660

[hoae017-B7] Baker P. Review of the National Men’s Health Policy and Action Plan 2008-2013: Final Report for the Health Service Executive. 2015. http://pbmenshealth.co.uk/wp-content/uploads/2015/05/Ireland-Mens-Health-Policy-Review.Final-Full-Report.2015.pdf

[hoae017-B8] Baker P. From the Margins to the Mainstream: Advocating the Inclusion of Men’s Health in Policy. A Scoping Study. London: Global Action on Men’s Health, 2020. https://gamh.org/wp-content/uploads/2020/06/From-the-Margins-to-The-Mainstream-Report.pdf

[hoae017-B9] Baker P , White A, Morgan R. Men’s health: COVID-19 pandemic highlights need for overdue policy action. Lancet 2020;395:1886–1888. 32563357 10.1016/S0140-6736(20)31303-9 PMC7836892

[hoae017-B10] Balbach M , Rossetti T, Ferreira J, Ghanem L, Ritagliati C, Myers RW, Huggins DJ, Steegborn C, Miranda IC, Meinke PT et al On-demand male contraception via acute inhibition of soluble adenylyl cyclase. Nat Commun 2023;14:637. 36788210 10.1038/s41467-023-36119-6 PMC9929232

[hoae017-B11] Barazani Y , Katz BF, Nagler HM, Stember DS. Lifestyle, environment, and male reproductive health. Urol Clin North Am 2014;41:55–66. 24286767 10.1016/j.ucl.2013.08.017

[hoae017-B12] Barratt CLR , De Jonge CJ, Anderson RA, Eisenberg ML, Garrido N, Rautakallio Hokkanen S, Krausz C, Kimmins S, O’Bryan MK, Pacey AA et al A global approach to addressing the policy, research and social challenges of male reproductive health. Hum Reprod Open 2021;2021:hoab009. 33768166 10.1093/hropen/hoab009 PMC7982782

[hoae017-B13] Batty GD , Mortensen LH, Shipley MJ. Semen quality and risk factors for mortality. Epidemiology 2019;30:e19–e21. 30640218 10.1097/EDE.0000000000000968

[hoae017-B14] Behboudi-Gandevani S , Bidhendi-Yarandi R, Panahi MH, Vaismoradi M. A systematic review and meta-analysis of male infertility and the subsequent risk of cancer. Front Oncol 2021;11:696702. 34722244 10.3389/fonc.2021.696702 PMC8551623

[hoae017-B15] Behre HM , Zitzmann M, Anderson RA, Handelsman DJ, Lestari SW, McLachlan RI, Meriggiola MC, Misro MM, Noe G, Wu FC et al Efficacy and safety of an injectable combination hormonal contraceptive for men. J Clin Endocrinol Metab 2016;101:4779–4788. 27788052 10.1210/jc.2016-2141

[hoae017-B16] Belladelli F , Muncey W, Eisenberg ML. Reproduction as a window for health in men. Fertil Steril 2023;120:429–437. 36642302 10.1016/j.fertnstert.2023.01.014

[hoae017-B17] Björndahl L , Barratt CLR, Mortimer D, Agarwal A, Aitken RJ, Alvarez JG, Aneck-Hahn N, Arver S, Baldi E, Bassas L et al Standards in semen examination: publishing reproducible and reliable data based on high-quality methodology. Hum Reprod 2022;37:2497–2502. 36112046 10.1093/humrep/deac189 PMC9627864

[hoae017-B18] Bole R , Lundy SD, Pei E, Bajic P, Parekh N, Vij SC. Rising vasectomy volume following reversal of federal protections for abortion rights in the United States. Int J Impot Res 2023;1–4. 10.1038/s41443-023-00672-x PMC9925925 36788351

[hoae017-B19] Bonde JP , Ernst E, Jensen TK, Hjollund NH, Kolstad H, Henriksen TB, Scheike T, Giwercman A, Olsen J, Skakkebaek NE. Relation between semen quality and fertility: a population-based study of 430 first-pregnancy planners. Lancet 1998;352:1172–1177. 9777833 10.1016/S0140-6736(97)10514-1

[hoae017-B20] CDC (Centers for Disease Control and Prevention). 2020 Assisted Reproductive Technology Fertility Clinic and National Summary Report. US Dept of Health and Human Services, 2022. https://www.cdc.gov/art/reports/2020/pdf/report-art-fertility-clinic-national-summary-h.pdf

[hoae017-B21] Chambers GM , Dyer S, Zegers-Hochschild F, de Mouzon J, Ishihara O, Banker M, Mansour R, Kupka MS, Adamson GD. International Committee for Monitoring Assisted Reproductive Technologies world report: assisted reproductive technology, 2014. Hum Reprod 2021;36:2921–2934. 34601605 10.1093/humrep/deab198

[hoae017-B51] Chen P , Li F, Harmer P. Healthy China 2030: moving from blueprint to action with a new focus on public health. Lancet Public Health 2019;4:e447. 31493840 10.1016/S2468-2667(19)30160-4

[hoae017-B22] Cipriani S , Ricci E, Chiaffarino F, Esposito G, Dalmartello M, La Vecchia C, Negri E, Parazzini F. Trend of change of sperm count and concentration over the last two decades: a systematic review and meta-regression analysis. Andrology 2023;11:997–1008. 36709405 10.1111/andr.13396

[hoae017-B23] Clemence M , King L. IPSOS Veracity Index 2022. Ipsos Mori, 2022. https://www.ipsos.com/sites/default/files/ct/news/documents/2022-11/Veracity%20index%202022_v2_PUBLIC.pdf

[hoae017-B24] Colagross-Schouten A , Lemoy MJ, Keesler RI, Lissner E, VandeVoort CA. The contraceptive efficacy of intravas injection of Vasalgel™ for adult male rhesus monkeys. Basic Clin Androl 2017;27:4. 28191316 10.1186/s12610-017-0048-9 PMC5294830

[hoae017-B25] Commonwealth of Australia (Department of Health). National Men’s Health Strategy 2020-2030. Canberra: Creative Commons License, 2019. https://www.health.gov.au/resources/publications/national-mens-health-strategy-2020-2030 (29 April 2024, date last accessed).

[hoae017-B26] Connolly MP , Pollard MS, Hoorens S, Kaplan BR, Oskowitz SP, Silber SJ. Long-term economic benefits attributed to IVF-conceived children: a lifetime tax calculation. Am J Manag Care 2008;14:598–604. 18778175

[hoae017-B116] Craig S , Jessop C, Perera P, Greenwood E. Wellcome Monitor 2020 How the British public engage with health research. Prepared for: Wellcome, 2021. https://cms.wellcome.org/sites/default/files/2021-02/wellcome-monitor-2020-public-engagement_0.pdf

[hoae017-B27] Daumler D , Chan P, Lo KC, Takefman J, Zelkowitz P. Men’s knowledge of their own fertility: a population-based survey examining the awareness of factors that are associated with male infertility. Hum Reprod 2016;31:2781–2790. 27816924 10.1093/humrep/dew265 PMC5193328

[hoae017-B28] De Jonge C , Barratt CLR. The present crisis in male reproductive health: an urgent need for a political, social, and research roadmap. Andrology 2019;7:762–768. 31241256 10.1111/andr.12673

[hoae017-B29] De Jonge CJ , Barratt CLR, Pacey AA. Counting the hidden costs of male reproductive health. World J Mens Health 2022;40:344–345. 35021301 10.5534/wjmh.210181 PMC8987135

[hoae017-B30] De Jonge CJ , Gellatly SA, Vazquez-Levin MH, Barratt CLR, Rautakallio-Hokkanen S. Male attitudes towards infertility: results from a global questionnaire. World J Mens Health 2023;41:204–214. 36047077 10.5534/wjmh.220099 PMC9826912

[hoae017-B31] de Visser RO , Richters J, Rissel C, Badcock PB, Simpson JM, Smith AM, Grulich AE. Change and stasis in sexual health and relationships: comparisons between the First and Second Australian Studies of Health and Relationships. Sex Health 2014;11:505–509. 25377003 10.1071/SH14112

[hoae017-B32] Díaz-Rojas D-I , Guerrero-Parra N-C, Robles-Carreño M-I, Rodríguez-Medina J, Lafaurie-Villamil M-M. Hombres, salud sexual y salud reproductiva: avances de la investigación reciente en América Latina. Men, sexual health and reproductive health: recent research advances in Latin America. Rev Colomb Enferm 2020;19:e021.

[hoae017-B33] Dupree JM , Dickey RM, Lipshultz LI. Inequity between male and female coverage in state infertility laws. Fertil Steril 2016;105:1519–1522. 26953734 10.1016/j.fertnstert.2016.02.025

[hoae017-B34] Eisenberg ML , Lathi RB, Baker VL, Westphal LM, Milki AA, Nangia AK. Frequency of the male infertility evaluation: data from the national survey of family growth. J Urol 2013;189:1030–1034. 23009868 10.1016/j.juro.2012.08.239

[hoae017-B35] Eisenberg ML , Li S, Behr B, Pera RR, Cullen MR. Relationship between semen production and medical comorbidity. Fertil Steril2015a;103:66–71. 25497466 10.1016/j.fertnstert.2014.10.017

[hoae017-B36] Eisenberg ML , Li S, Brooks JD, Cullen MR, Baker LC. Increased risk of cancer in infertile men: analysis of U.S. claims data. J Urol2015b;193:1596–1601. 25463997 10.1016/j.juro.2014.11.080

[hoae017-B37] Erkek S , Hisano M, Liang CY, Gill M, Murr R, Dieker J, Schübeler D, van der Vlag J, Stadler MB, Peters AH. Molecular determinants of nucleosome retention at CpG-rich sequences in mouse spermatozoa. Nat Struct Mol Biol 2013;20:868–875. 23770822 10.1038/nsmb.2599

[hoae017-B38] European Atlas for Fertility Treatment. 2021. https://fertilityeurope.eu/wp-content/uploads/2021/12/FERTIL-Atlas_EN-2021-v10.pdf (29 April 2024, date last accessed).

[hoae017-B111] European Commission, Directorate-General for Communication, The EU in 2016: general report on the activities of the European Union. Publications Office of the European Union; 2017. doi/10.2775/226192.

[hoae017-B39] European IVF Monitoring Consortium (EIM), for the European Society of Human Reproduction and Embryology (ESHRE); Smeenk J, Wyns C, De Geyter C, Kupka M, Bergh C, Cuevas Saiz I, De Neubourg D, Rezabek K, Tandler-Schneider A, Rugescu I, et al ART in Europe, 2018: results generated from European registries by ESHRE. Hum Reprod 2022;38:2321–2338.

[hoae017-B40] Fauser BC , Bouchard P, Coelingh Bennink HJ, Collins JA, Devroey P, Evers JL, van Steirteghem A. Alternative approaches in IVF. Hum Reprod Update 2002;8:1–9. 11871382 10.1093/humupd/8.1.1

[hoae017-B41] Fauser BCJM , Adamson GD, Boivin J, Chambers GM, de Geyter C, Dyer S, Inhorn MC, Schmidt L, Serour GI, Tarlatzis B, et al; IFFS Demographics and Access to Care Review Board. Declining global fertility rates and the implications for family planning and family building: an IFFS consensus document based on a narrative review of the literature. Hum Reprod Update 2024;30:153–173. 38197291 10.1093/humupd/dmad028 PMC10905510

[hoae017-B42] Fitz-James MH , Cavalli G. Molecular mechanisms of transgenerational epigenetic inheritance. Nat Rev Genet 2022;23:325–341. 34983971 10.1038/s41576-021-00438-5 PMC7619059

[hoae017-B43] Frank D , Kufa T, Dorrell P, Kularatne R, Maithufi R, Chidarikire T, Pillay Y, Mokgatle M. Evaluation of the national clinical sentinel surveillance system for sexually transmitted infections in South Africa: analysis of provincial and district-level data. S Afr Med J 2023;113:41–48. 10.7196/SAMJ.2023.v113i7.365 37882040

[hoae017-B44] Gavin M , Hausmann R, Lora R, Pagés C, Sauedoff WD, Szekely M, Westley GD. Facing Up to Inequality in Latin America. Economic And Social Progress In Latin America 1998-1999 Report. Baltimore, Maryland, USA: The Johns Hopkins University Press, 1998.

[hoae017-B45] Gimenes F , Souza RP, Bento JC, Teixeira JJ, Maria-Engler SS, Bonini MG, Consolaro ME. Male infertility: a public health issue caused by sexually transmitted pathogens. Nat Rev Urol 2014;11:672–687. 25330794 10.1038/nrurol.2014.285

[hoae017-B46] Global Health 50:50. 2021. https://globalhealth5050.org/the-sex-gender-and-covid-19-project/ (29 April 2024, date last accessed).

[hoae017-B47] Goodyear V , Andersson J, Quennerstedt M, Varea V. #Gymlad—young boys learning processes and health-related social media. Qual Res Sport Exerc Health 2022;14:1–33. 32166044 10.1080/2159676X.2019.1673470 PMC7034328

[hoae017-B48] Gumerova E , De Jonge CJ, Barratt CLR. Research funding for male reproductive health and infertility in the UK and USA [2016–2019]. Hum Fertil (Camb) 2023;26:439–449. 35302424 10.1080/14647273.2022.2045521

[hoae017-B49] Hammarberg K , Collins V, Holden C, Young K, McLachlan R. Men’s knowledge, attitudes and behaviours relating to fertility. Hum Reprod Update 2017;23:458–480. 28333354 10.1093/humupd/dmx005

[hoae017-B50] Hansen LS , Priskorn L, Holmboe SA, Jensen TK, Hansen AH, Andersson AM, Jørgensen N. Testicular function is associated with cardiometabolic health markers: a cross-sectional study of 2289 young men. Andrology 2023;11:561–574. 36520458 10.1111/andr.13365

[hoae017-B52] Healthy Male. Plus Paternal: A Focus on Fathers Case for Change. Melbourne: Healthy Male, 2020. https://www.healthymale.org.au/files/inline-files/Healthy%20Male_Plus%20Paternal_Case%20for%20change_Full%20report_Final%20%5BScreen%20reader%20accessible%5D_1.pdf

[hoae017-B53] Houston BJ , Riera-Escamilla A, Wyrwoll MJ, Salas-Huetos A, Xavier MJ, Nagirnaja L, Friedrich C, Conrad DF, Aston KI, Krausz C et al A systematic review of the validated monogenic causes of human male infertility: 2020 update and a discussion of emerging gene-disease relationships. Hum Reprod Update 2021;28:15–29. 34498060 10.1093/humupd/dmab030 PMC8730311

[hoae017-B54] Ilic M , Ilic I. Worldwide suicide mortality trends (2000-2019): a joinpoint regression analysis. World J Psychiatry 2022;12:1044–1060. 36158305 10.5498/wjp.v12.i8.1044 PMC9476842

[hoae017-B55] Japan Family Planning Association. The 8th danjyo no seikatsu to ishiki ni kansuru chosa (National Lifestyle and Attitudes Towards Sexual Behavior Survey). Japan Family Planning Association, 2017.

[hoae017-B56] Jimbo M , Kunisaki J, Ghaed M, Yu V, Flores HA, Hotaling JM. Fertility in the aging male: a systematic review. Fertil Steril 2022;118:1022–1034. 36509505 10.1016/j.fertnstert.2022.10.035 PMC10914128

[hoae017-B57] Jørgensen N , Lamb DJ, Levine H, Pastuszak AW, Sigalos JT, Swan SH, Eisenberg ML. Are worldwide sperm counts declining? Fertil Steril 2021;116:1457–1463. 34836581 10.1016/j.fertnstert.2021.10.020

[hoae017-B58] Kaati G , Bygren LO, Pembrey M, Sjöström M. Transgenerational response to nutrition, early life circumstances and longevity. Eur J Hum Genet 2007;15:784–790. 17457370 10.1038/sj.ejhg.5201832

[hoae017-B59] Keihani S , Verrilli LE, Zhang C, Presson AP, Hanson HA, Pastuszak AW, Johnstone EB, Hotaling JM. Semen parameter thresholds and time-to-conception in subfertile couples: how high is high enough? Hum Reprod 2021;36:2121–2133. 34097024 10.1093/humrep/deab133 PMC8660554

[hoae017-B60] Kimmins S , Anderson RA, Barratt CLR, Behre HM, Catford SR, De Jonge CJ, Delbes G, Eisenberg ML, Garrido N, Houston BJ et al Frequency, morbidity and equity—the case for increased research on male fertility. Nat Rev Urol 2024;21:102–124. 37828407 10.1038/s41585-023-00820-4

[hoae017-B61] Kimmins S , Sassone-Corsi P. Chromatin remodelling and epigenetic features of germ cells. Nature 2005;434:583–589. 15800613 10.1038/nature03368

[hoae017-B62] Kontula O. Sex life challenges: the Finnish case. In: Wright JD (ed). International Encyclopedia of the Social & Behavioral Sciences, 2nd edn. Oxford, UK: Elsevier, 2015,665–671.

[hoae017-B63] Krausz C , Riera-Escamilla A, Moreno-Mendoza D, Holleman K, Cioppi F, Algaba F, Pybus M, Friedrich C, Wyrwoll MJ, Casamonti E et al Genetic dissection of spermatogenic arrest through exome analysis: clinical implications for the management of azoospermic men. Genet Med 2020;22:1956–1966. 32741963 10.1038/s41436-020-0907-1 PMC7710580

[hoae017-B64] Lambrot R , Chan D, Shao X, Aarabi M, Kwan T, Bourque G, Moskovtsev S, Librach C, Trasler J, Dumeaux V et al Whole-genome sequencing of H3K4me3 and DNA methylation in human sperm reveals regions of overlap linked to fertility and development. Cell Rep 2021;36:109418. 34289352 10.1016/j.celrep.2021.109418

[hoae017-B65] Latif T , Kold Jensen T, Mehlsen J, Holmboe SA, Brinth L, Pors K, Skouby SO, Jørgensen N, Lindahl-Jacobsen R. Semen quality as a predictor of subsequent morbidity: a Danish Cohort Study of 4,712 men with long-term follow-up. Am J Epidemiol 2017;186:910–917. 28498890 10.1093/aje/kwx067

[hoae017-B66] Latif T , Lindahl-Jacobsen R, Mehlsen J, Eisenberg ML, Holmboe SA, Pors K, Brinth L, Skouby SO, Jørgensen N, Jensen TK. Semen quality associated with subsequent hospitalizations—can the effect be explained by socio-economic status and lifestyle factors? Andrology 2018;6:428–435. 29481730 10.1111/andr.12477

[hoae017-B67] Lee RD , Mason A. Population Aging and the Generational Economy: A Global Perspective. Northampton, MA, USA: Edward Elgar Publishing, 2011.

[hoae017-B68] Legese N , Tura AK, Roba KT, Demeke H. The prevalence of infertility and factors associated with infertility in Ethiopia: analysis of Ethiopian Demographic and Health Survey (EDHS). PLoS One 2023;18:e0291912. 37824486 10.1371/journal.pone.0291912 PMC10569515

[hoae017-B69] Levine H , Jørgensen N, Martino-Andrade A, Mendiola J, Weksler-Derri D, Jolles M, Pinotti R, Swan SH. Temporal trends in sperm count: a systematic review and meta-regression analysis of samples collected globally in the 20th and 21st centuries. Hum Reprod Update 2023;29:157–176. 36377604 10.1093/humupd/dmac035

[hoae017-B70] Liao SJ , Xu YY, Sun RJ, Lyu QY. The development of basic research in the field of male reproductive health in China over the past 30 years supported by the National Natural Science Foundation of China. National Journal of Andrology Zhonghua Nan Ke Xue Za Zhi 2020;26:3–16. 33345471

[hoae017-B71] Lismer A , Dumeaux V, Lafleur C, Lambrot R, Brind’Amour J, Lorincz MC, Kimmins S. Histone H3 lysine 4 trimethylation in sperm is transmitted to the embryo and associated with diet-induced phenotypes in the offspring. Dev Cell 2021;56:671–686.e6. 33596408 10.1016/j.devcel.2021.01.014

[hoae017-B72] Lismer A , Kimmins S. Emerging evidence that the mammalian sperm epigenome serves as a template for embryo development. Nat Commun 2023;14:2142. 37059740 10.1038/s41467-023-37820-2 PMC10104880

[hoae017-B73] Ly L , Chan D, Aarabi M, Landry M, Behan NA, MacFarlane AJ, Trasler J. Intergenerational impact of paternal lifetime exposures to both folic acid deficiency and supplementation on reproductive outcomes and imprinted gene methylation. Mol Hum Reprod 2017;23:461–477. 28535307 10.1093/molehr/gax029 PMC5909862

[hoae017-B74] Madsen JB , Moslehi S, Wang C. What has driven the great fertility decline in developing countries since 1960? Journal of Development Studies 2018;54:738–757.

[hoae017-B75] Malina A , Głogiewicz M, Piotrowski J. Supportive social interactions in infertility treatment decrease cortisol levels: experimental study report. Front Psychol 2019;10:2779. 31920828 10.3389/fpsyg.2019.02779 PMC6927458

[hoae017-B76] Mariani NAP , Silva JV, Fardilha M, Silva EJR. Advances in non-hormonal male contraception targeting sperm motility. Hum Reprod Update 2023;29:545–569. 37141450 10.1093/humupd/dmad008

[hoae017-B77] McNaughton-Cassill ME , Bostwick JM, Vanscoy SE, Arthur NJ, Hickman TN, Robinson RD, Neal GS. Development of brief stress management support groups for couples undergoing in vitro fertilization treatment. Fertil Steril 2000;74:87–93. Erratum in: *Fertil Steril* 2000;74:851. Bostwick M [corrected to Bostwick JM]. 10899502 10.1016/s0015-0282(00)00564-1

[hoae017-B78] Mehta A , Nangia AK, Dupree JM, Smith JF. Limitations and barriers in access to care for male factor infertility. Fertil Steril 2016;105:1128–1137. 27054307 10.1016/j.fertnstert.2016.03.023

[hoae017-B79] Mercer CH , Tanton C, Prah P, Erens B, Sonnenberg P, Clifton S, Macdowall W, Lewis R, Field N, Datta J et al Changes in sexual attitudes and lifestyles in Britain through the life course and over time: findings from the National Surveys of Sexual Attitudes and Lifestyles (Natsal). Lancet 2013;382:1781–1794. 24286784 10.1016/S0140-6736(13)62035-8 PMC3899021

[hoae017-B80] Miner MM , Heidelbaugh J, Paulos M, Seftel AD, Jameson J, Kaplan SA. The intersection of medicine and urology: an emerging paradign of sexual function, cardiometabolic risk, bone health and men’s health centers. Med Clin North Am 2018;102:399–415. 29406067 10.1016/j.mcna.2017.11.002

[hoae017-B81] Mommers E , Kersemaekers WM, Elliesen J, Kepers M, Apter D, Behre HM, Beynon J, Bouloux PM, Costantino A, Gerbershagen HP et al Male hormonal contraception: a double-blind, placebo-controlled study. J Clin Endocrinol Metab 2008;93:2572–2580. 18413423 10.1210/jc.2008-0265

[hoae017-B82] Najari BB. The demographics of men presenting to male factor infertility specialists: the impressive first report from the Andrology Research Consortium. Fertil Steril 2019;112:642–643. 31561868 10.1016/j.fertnstert.2019.07.004

[hoae017-B83] Nickels L , Yan W. Nonhormonal male contraceptive development-strategies for progress. Pharmacol Rev 2023;76:37–48. 38101934 10.1124/pharmrev.122.000787 PMC10759220

[hoae017-B84] Nya-Ngatchou JJ , Amory JK. New approaches to male non-hormonal contraception. Contraception 2013;87:296–299. 22995542 10.1016/j.contraception.2012.08.016 PMC3529759

[hoae017-B85] Ombelet W , Van Blerkom J, Bruckers L, Dhont N, Nargund G, Campo R. Promising perinatal outcome after using a simplified low-cost IVF culture system specifically designed for resource-poor countries. J Clin Med 2023;12:2264. 36983264 10.3390/jcm12062264 PMC10059708

[hoae017-B86] Ombelet W , Van Blerkom J, Nargund G, Van der Auwera I, Janssen M, Dhont N, Bosmans E, Boshoff G, Vertessen VJ, Campo R. Multiyear outcomes using sibling oocytes demonstrates safety and efficacy of a simplified culture system consistent with use in a low-cost IVF setting. Reprod Biomed Online 2022;45:481–490. 36064261 10.1016/j.rbmo.2022.04.008

[hoae017-B87] Oud MS , Smits RM, Smith HE, Mastrorosa FK, Holt GS, Houston BJ, de Vries PF, Alobaidi BKS, Batty LE, Ismail H, et al; Genetics of Male Infertility Initiative (GEMINI) Consortium. A de novo paradigm for male infertility. Nat Commun. 2022;13:154. 35013161 10.1038/s41467-021-27132-8 PMC8748898

[hoae017-B88] Oud MS , Volozonoka L, Smits RM, Vissers LELM, Ramos L, Veltman JA. A systematic review and standardized clinical validity assessment of male infertility genes. Hum Reprod 2019;34:932–941. 30865283 10.1093/humrep/dez022 PMC6505449

[hoae017-B89] PAHO. Strategic Plan of the Pan American Health Organization 2020-2025: Equity at the Heart of Health. (Official Document: 359) Pan American Health Organization, 2020.

[hoae017-B90] Pembrey ME , Bygren LO, Kaati G, Edvinsson S, Northstone K, Sjöström M, Golding J; ALSPAC Study Team. Sex-specific, male-line transgenerational responses in humans. Eur J Hum Genet 2006;14:159–166. 16391557 10.1038/sj.ejhg.5201538

[hoae017-B91] Qiao J , Wang Y, Li X, Jiang F, Zhang Y, Ma J, Song Y, Ma J, Fu W, Pang R et al A Lancet Commission on 70 years of women’s reproductive, maternal, newborn, child, and adolescent health in China. Lancet 2021;397:2497–2536. 34043953 10.1016/S0140-6736(20)32708-2

[hoae017-B92] Reynolds-Wright JJ , Anderson R. Male contraception: where are we going and where have we been? BMJ Sex Reprod Health 2019;45:236–242. Erratum in: *BMJ Sex Reprod Health* 2020;46:157. 10.1136/bmjsrh-2019-200395 PMC6892591 31537614

[hoae017-B93] Reynolds-Wright JJ , Cameron NJ, Anderson RA. Will men use novel male contraceptive methods and will women trust them? A systematic review. J Sex Res 2021;58:838–849. 33900134 10.1080/00224499.2021.1905764

[hoae017-B94] Ricci E , Al Beitawi S, Cipriani S, Candiani M, Chiaffarino F, Viganò P, Noli S, Parazzini F. Semen quality and alcohol intake: a systematic review and meta-analysis. Reprod Biomed Online 2017;34:38–47. 28029592 10.1016/j.rbmo.2016.09.012

[hoae017-B95] Rimmer MP , Howie RA, Subramanian V, Anderson RA, Bertolla RP, Beebeejaun Y, Bortoletto P, Sunkara SK, Mitchell RT, Pacey A et al Outcome reporting across randomized controlled trials evaluating potential treatments for male infertility: a systematic review. Hum Reprod Open 2022;2022:hoac010. 35386119 10.1093/hropen/hoac010 PMC8982407

[hoae017-B96] Rizza P , Tommasino P. Do we treat future generations fairly? Italian fiscal policy through the prism of generational accounting. Giornale Degli Economisti e Annali di Economia 2010;69:115–153.

[hoae017-B97] Rosa-Villagrán L , Barrera N, Montes J, Riso C, Sapiro R. Decline of semen quality over the last 30 years in Uruguay. Basic Clin Androl 2021;31:8. 33952196 10.1186/s12610-021-00128-6 PMC8101031

[hoae017-B98] Sanchez-Molano SA , Forero-Martinez LJ, Rivillas-Garcia JC. Vasectomy in Colombia: how to adapt healthcare services to the needs of men? Rev. Fac. Nac. Salud Pública [Online] 2019;37:66–77.

[hoae017-B99] Sarkodie SA. The invisible hand and EKC hypothesis: what are the drivers of environmental degradation and pollution in Africa? Environ Sci Pollut Res Int 2018;25:21993–22022. 29797200 10.1007/s11356-018-2347-x

[hoae017-B100] Schlegel PN , Sigman M, Collura B, De Jonge CJ, Eisenberg ML, Lamb DJ, Mulhall JP, Niederberger C, Sandlow JI, Sokol RZ et al Diagnosis and treatment of infertility in men: AUA/ASRM guideline part I. Fertil Steril2021a;115:54–61. 33309062 10.1016/j.fertnstert.2020.11.015

[hoae017-B101] Schlegel PN , Sigman M, Collura B, De Jonge CJ, Eisenberg ML, Lamb DJ, Mulhall JP, Niederberger C, Sandlow JI, Sokol RZ et al Diagnosis and treatment of infertility in men: AUA/ASRM guideline part II. Fertil Steril2021b;115:62–69. 33309061 10.1016/j.fertnstert.2020.11.016

[hoae017-B102] Serour GI , Serour AG, El Faysal Y, Islam Y. The place of ART in Africa. Global Reproductive Health 2019;4:e 27.

[hoae017-B103] Serour GI , Serour AG. The impact of religion and culture on medically assisted reproduction in the Middle East and Europe. Reprod Biomed Online 2021;43:421–433. 34344602 10.1016/j.rbmo.2021.06.002

[hoae017-B104] Shand T , Marcell AV. Engaging men in sexual and reproductive health. In: McQueen DV, editor. Oxford research encyclopedia of global public health. New York: Oxford University Press, 2021. 10.1093/acrefore/9780190632366.013.215.

[hoae017-B105] Siklenka K , Erkek S, Godmann M, Lambrot R, McGraw S, Lafleur C, Cohen T, Xia J, Suderman M, Hallett M et al Disruption of histone methylation in developing sperm impairs offspring health transgenerationally. Science 2015;350:aab2006. 26449473 10.1126/science.aab2006

[hoae017-B106] Siqueira S , Ropelle AC, Nascimento JAA, Fazano FAT, Bahamondes LG, Gabiatti JR, Costa-Paiva L, Baccaro LF. Changes in seminal parameters among Brazilian men between 1995 and 2018. Sci Rep 2020;10:6430. 32286479 10.1038/s41598-020-63468-9 PMC7156660

[hoae017-B107] Sobotka T , Matysiak A, Brzozowska Z. Policy responses to low fertility: how effective are they? United Nations Population Fund 2019. https://www.unfpa.org/publications/policy-responses-low-fertility-how-effective-are-they

[hoae017-B108] Soleimani R , Ansari F, Hamzehgardeshi Z, Elyasi F, Moosazadeh M, Yazdani F, Shahidi M, Shiraghaei N, Karimi M, Hemati T et al Perceived stress reduction through an infertility coaching program: a randomized controlled clinical trial. Sci Rep 2023;13:14511. 37666933 10.1038/s41598-023-41845-4 PMC10477300

[hoae017-B109] Stone L. Pro-Natal Policies Work, But They Come with a Hefty Price Tag. Institute for Family Studies: IFS, 2020. https://ifstudies.org/blog/pro-natal-policies-work-but-they-come-with-a-hefty-price-tag (29 April 2024, date last accessed).

[hoae017-B110] Sung H , Ferlay J, Siegel RL, Laversanne M, Soerjomataram I, Jemal A, Bray F. Global Cancer Statistics 2020: GLOBOCAN estimates of incidence and mortality worldwide for 36 cancers in 185 countries. CA Cancer J Clin 2021;71:209–249. 33538338 10.3322/caac.21660

[hoae017-B112] Twenge JM. Possible reasons US adults are not having sex as much as they used to. JAMA Netw Open 2020;3:e203889. 32530467 10.1001/jamanetworkopen.2020.3889

[hoae017-B113] Ueda P , Mercer CH, Ghaznavi C, Herbenick D. Trends in frequency of sexual activity and number of sexual partners among adults aged 18 to 44 years in the US, 2000-2018. JAMA Netw Open 2020;3:e203833. 32530470 10.1001/jamanetworkopen.2020.3833 PMC7293001

[hoae017-B114] Vallejo-Ortega MT , Gaitán Duarte H, Mello MB, Caffe S, Perez F. A systematic review of the prevalence of selected sexually transmitted infections in young people in Latin America. Rev Panam Salud Publica 2022;46:e73. 35747471 10.26633/RPSP.2022.73 PMC9211030

[hoae017-B115] Verón GL , Tissera AD, Bello R, Beltramone F, Estofan G, Molina RI, Vazquez-Levin MH. Impact of age, clinical conditions, and lifestyle on routine semen parameters and sperm kinematics. Fertil Steril 2018;110:68–75.e4. 29980266 10.1016/j.fertnstert.2018.03.016

[hoae017-B117] WHO. Contraceptive efficacy of testosterone-induced azoospermia in normal men. World Health Organization Task Force on methods for the regulation of male fertility. Lancet 1990;336:955–959. 1977002

[hoae017-B118] World Health Organization. Regional Office for Europe.The health and well-being of men in the WHO European Region: better health through a gender approach.World Health Organization. Regional Office for Europe, 2018. https://iris.who.int/handle/10665/329686 (29 April 2024, date last accessed).

[hoae017-B119] WHO. World Health Statistics 2019: Monitoring Health for the SDGs, Sustainable Development Goals. Geneva: World Health Organization, 2019.

[hoae017-B120] WHO. World Health Statistics 2023: Monitoring Health for the SDGs, Sustainable Development Goals. Geneva: WHO, 2023a.

[hoae017-B121] WHO. Infertility Prevalence Estimates, 1990–2021. Geneva: World Health Organization, 2023b.

[hoae017-B122] Yu B , Zhang CA, Chen T, Mulloy E, Shaw GM, Eisenberg ML. Congenital male genital malformations and paternal health: an analysis of the US claims data. Andrology. 2023;11:1114–1120. 36727635 10.1111/andr.13404

