# Supplementary material for: Current global status of male reproductive health
Source: Hum Reprod Open. 2024 Apr 12;2024(2):hoae017. doi: 10.1093/hropen/hoae017 (PMC11065475; doi:10.1093/hropen/hoae017)
Supplement: hoae017_Supplementary_Data_File_S1 [file hoae017_supplementary_data_file_s1.docx]

**Supplementary Data File S1**

**ESHRE-MRHI Course. Male Reproductive Health**

**Day 1. Thursday 06 October 2022**

**Opening Session**

*Chair Richard Anderson, United Kingdom*

Opening. Christopher Barratt, United Kingdom and Christopher De Jonge, USA

Welcome message. Zsotz Kopa, Hungary

WHO - SDG's and global improvement of men's reproductive health. James Kiarie, Switzerland

Male Reproductive Health Initiative Global Questionnaire. Results and moving forward. Christopher De Jonge, USA

**Session 1: Global status of Male Reproductive Health: SWOT Analysis**

*Chair Satu Rautakallio-Hokkanen, Belgium*

European Union. Nicolás Garrido Puchalt, Spain

South America. Mónica Vazquez-Levin, Argentina

North America. Sarah Kimmins, Canada

Africa. Gamal Serour, Egypt

Australasia. Robert McLachlan, Australia

China. David Yiu Leung Chan, Hong Kong

**Session 2: Global status of and threats to male reproductive health**

*Chair Michael Eisenberg, USA*

What are threats to MRH. Allan Pacey, United Kingdom

Population dynamics. Robert John Aitken, Australia

Next generation and beyond. Sarah Kimmins, Canada

**Session 3: HRU paper 2021 - Rallying support for male reproductive health**

*Chair Chris Barratt, United Kingdom*

Questions. Moira K O'Bryan, Australia

How to address the questions. Joris Veltman, United Kingdom

Contraception. Richard Anderson, United Kingdom

Medically assisted reproduction. Michael Eisenberg, USA.

Public information, interaction with patients. Allan Pacey, United Kingdom

**Day 2. Friday 07 October 2022**

**Session 4: Networking, coordination, and achievement in other arenas**

*Chair Mónica Vazquez-Levin, Argentina*

A report from global action on men's health. Peter Baker, United Kingdom

The economic consequences of unmet need in assisted reproduction. Mark Connolly, USA.

Policy makers and how to influence change: Fertility Europe. Satu Rautakallio Hokkanen, Belgium

Policy makers and how to influence change: Resolve. Barbara Collura, U.S.A.

Effective policies that have changed reproductive health for women in Scotland. Corinne Love, United Kingdom

**Session 5: Conflict and solutions**

*Chair Nicolas Garrido Puchalt, Spain*

Be the future of Andrology. Donna Vogel, USA

Reproductive & somatic health - cousins? Michael Eisenberg, USA

Reproductive & somatic health - distant relatives? Niels Jorgensen, Denmark

Training of the current and next generation - status quo. Csilla Gabriella Krausz Italy

Training of the current and next generation - time to remodel? Craig Niederberger, USA.

**Session 6: Conclusions**

*Chair Christopher De Jonge, U.S.A.*

What are the conclusions for the way forward? Moira K O'Bryan, Australia

End of Workshop: Summary & Q/A. Allan Pacey, United Kingdom
