# Supplementary material for: Current global status of male reproductive health
Source: Hum Reprod Open. 2024 Apr 12;2024(2):hoae017. doi: 10.1093/hropen/hoae017 (PMC11065475; doi:10.1093/hropen/hoae017)
Supplement: hoae017_Supplementary_Data_File_S2 [file hoae017_supplementary_data_file_s2.docx]

**Supplementary Data File S2**

**United Nations Sustainable Development Goals (SDGs)***

**
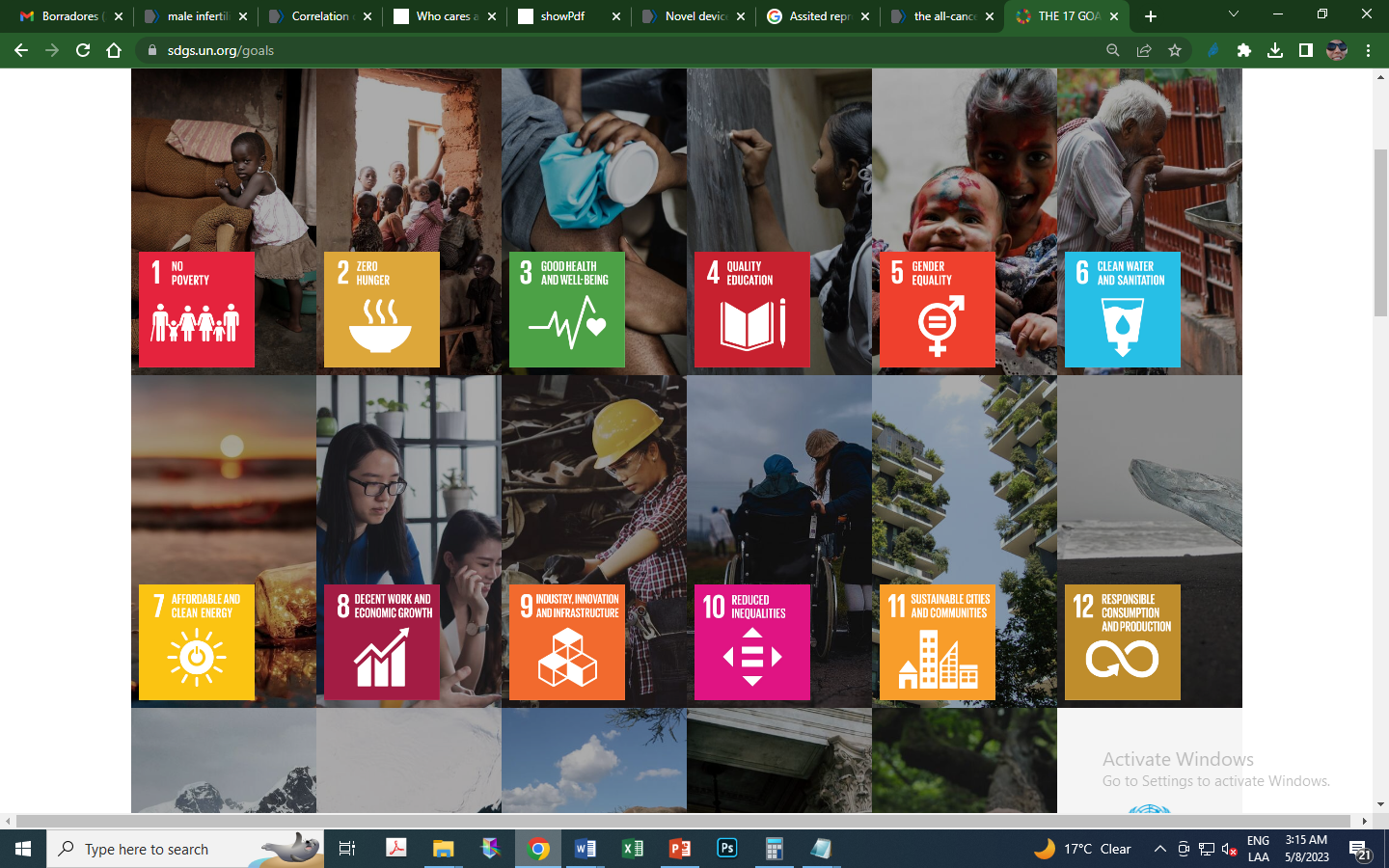
*** <https://sdgs.un.org/goals>

**
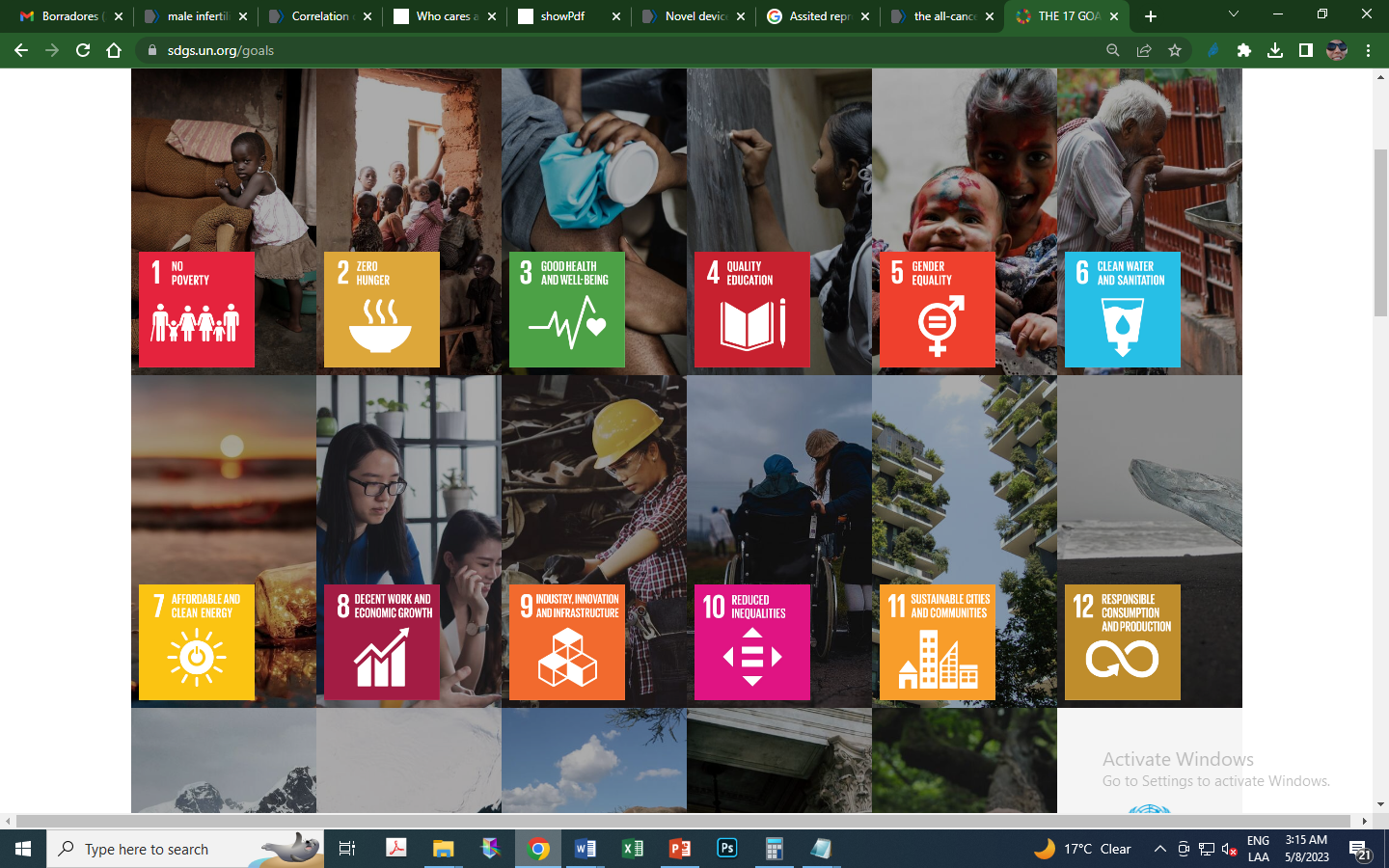
**

**
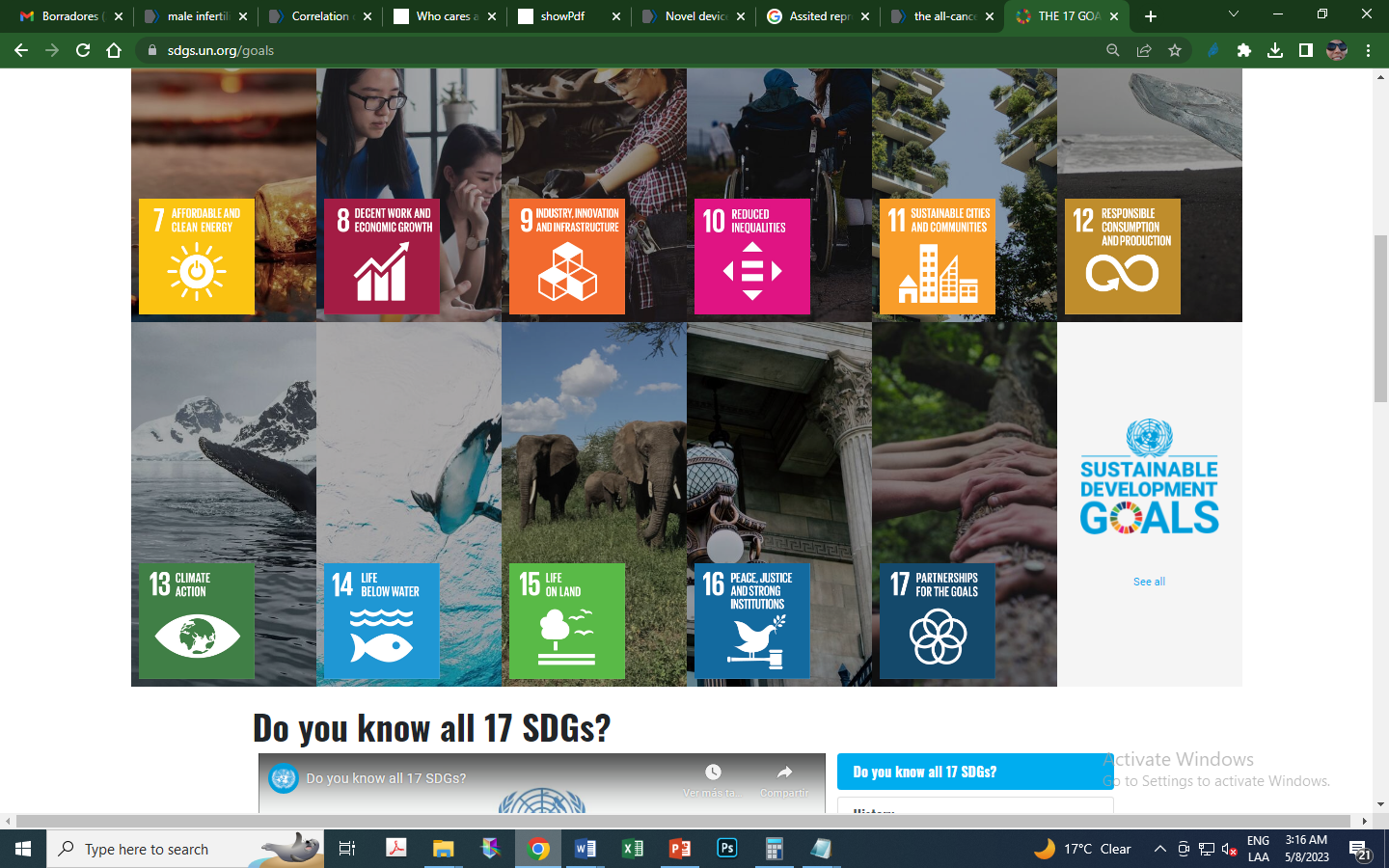
**

|  | **Goals** |
| --- | --- |
| **1** | End poverty in all its forms everywhere |
| **2** | End hunger, achieve food security and improved nutrition and promote sustainable agriculture |
| **3** | Ensure healthy lives and promote well-being for all at all ages |
| **4** | Ensure inclusive and equitable quality education and promote lifelong learning opportunities for all |
| **5** | Achieve gender equality and empower all women and girls |
| **6** | Ensure availability and sustainable management of water and sanitation for all |
| **7** | Ensure access to affordable, reliable, sustainable and modern energy for all |
| **8** | Promote sustained, inclusive and sustainable economic growth, full and productive employment and decent work for all |
| **9** | Build resilient infrastructure, promote inclusive and sustainable industrialization and foster innovation |
| **10** | Reduce inequality within and among countries |
| **11** | Make cities and human settlements inclusive, safe, resilient and sustainable |
| **12** | Ensure sustainable consumption and production patterns |
| **13** | Take urgent action to combat climate change and its impacts |
| **14** | Conserve and sustainably use the oceans, seas and marine resources for sustainable development |
| **15** | Protect, restore and promote sustainable use of terrestrial ecosystems, sustainably manage forests, combat desertification, and halt and reserve land degradation and halt biodiversity loss |
| **16** | Promote peaceful and inclusive societies for sustainable development, provide access to justice for all and build effective, accountable and inclusive institutions at all levels |
| **17** | Strengthen the means of implementation and revitalize the Global Partnership for Sustainable Development |
