# Supplementary material for: Current global status of male reproductive health
Source: Hum Reprod Open. 2024 Apr 12;2024(2):hoae017. doi: 10.1093/hropen/hoae017 (PMC11065475; doi:10.1093/hropen/hoae017)
Supplement: hoae017_Supplementary_Data_File_S3 [file hoae017_supplementary_data_file_s3.docx]

**Supplementary Data File S3**

**Be the Future of Andrology**

*Prof. Dr. Donna Vogel, MD PhD*

“…The organizers invited me to talk about leadership. But that really meant attracting people to our field. As you’ll see, these are closely related. I got into andrology when, during my MD-PhD studies in the 1970s, I participated in a summer course, Fertilization and Gamete Physiology, at the Marine Biological Lab in Woods Hole, Mass. One evening, I decided I could save the world by inventing the perfect male contraceptive. After completing my degrees and three years of residency, I did a combined postdoc and clinical fellowship in endocrinology. This involved basic and clinical research and patient care relating to male reproductive health and infertility. My first job, and the one I held the longest, was running a grant program at NIH. I was the Program Director for Reproductive Medicine for 13 years. In that role, I had a surprising degree of impact on the shape of the field. I would like to convince you that you can have an impact even if you take a somewhat unconventional career trajectory. This is especially true in andrology. Why? 1) Our field is relatively small. 2) Our membership covers a broad range of scientific and clinical disciplines. It’s hard to make an impact if a field is large and homogeneous. It’s easier when a field is small and diverse. Let’s dig into that a little.

Who is an andrologist? And is there only one kind of andrologist? While there are differences in how the word is used in Europe, the US, and elsewhere, I’m from the States and am used to a broad definition. That is, it’s not just the person in the clinic who tests or treats the male partner of an infertile couple. We are basic, clinical, translational, population, and behavioral scientists, including students and trainees. We are researchers, clinicians, technical staff, communicators, educators, advocates. Our fields include urology, endocrinology, gynecology, toxicology, immunology, pharmacology, and epidemiology. We work in academia, industry, government, nonprofit, and media - all over the world.

For these reasons, andrology is a field where you can make a difference. In research, innovation comes from bringing things together that haven’t been put together before: asking a question in a new way, using models or approaches that have worked in one system but haven’t been applied to your question before. In an organization, innovation comes from getting input from people with diverse backgrounds and experiences.

You may not think of yourself as a leader, or as someone who can make a difference. Why? Because you don’t see yourself as fitting the mold. Maybe you haven’t followed a “conventional” career path. Maybe you’re still early in your career. Maybe you’re not at what’s considered a major institution. Or you don’t have a PhD. Or you’re not in North America or Western Europe. Or you feel in some other way marginalized. However, because of everything we’ve been saying about andrology, you are in a better position to have an impact than you think. You know what Imposter Syndrome is (https://en.wikipedia.org/wiki/Impostor_syndrome) (Arakawa, 2022). I would give you the same advice I would give someone about overcoming Imposter Syndrome. Focus on what you have, and not on what’s missing. You have skill, experience, knowledge and talent. No matter who you are or where you are, you have something to contribute, even if it’s still a potential waiting to be tapped.

A former colleague of mine, Dr. Yvonne Maddox, gave a keynote on Health Disparities and Reproductive Sciences at the last meeting of the Society for the Study of Reproduction (SSR). She is now the head of a private foundation working to reduce disparities, especially in maternal health in minorities. SSR is not a clinical society. So why did Yvonne Maddox talk to the SSR? And why does that have anything to do with us today? Because she asserted that we need a reliable, credible basic science foundation for advances in clinical care. That is to say, we need basic reproductive research to advance equity in reproductive and sexual health.

The pandemic has presented many challenges to science. But there’s a silver lining. Learning, training, and mentoring going virtual has made it global. The ability to communicate and collaborate with anyone, anywhere enhances innovation and visibility.

Our field has many opportunities to make a contribution. As I wrote a few years ago in Andrology (Vogel, 2015), there are many paths to success, to becoming influential, which is to say, becoming a leader. What does leadership mean here today? A leader works with other people to make something happen. You may be a leader without seeing yourself that way, and you can become a leader in our field. Are you a leader? I have had an unconventional career path. I was never a professor. I haven’t touched a patient or done an experiment since 1987. But the American Society of Andrology elected me president, and I have a plaque on my wall to prove it.

Whether it’s through your research, your managerial skills, your communication skills, or something else, in andrology, it is easier than you may think to have an impact, to be a leader, to be the change. You may think you are not in a position to have impact. But you are, and you can…”

Arakawa S. Dare to Lead: How Administrators Can Overcome Impostor Syndrome. *The Chronicle of Higher Education* 2022. https://www.chronicle.com/article/dare-to-lead-how-administrators-can-overcome-impostor-syndrome

Vogel DL. Lessons in Andrology: many paths to success. *Andrology.* 2015; 3: 148-149.
