# Supplementary material for: Current global status of male reproductive health
Source: Hum Reprod Open. 2024 Apr 12;2024(2):hoae017. doi: 10.1093/hropen/hoae017 (PMC11065475; doi:10.1093/hropen/hoae017)
Supplement: hoae017_Supplementary_Table_S1 [file hoae017_supplementary_table_s1.docx]

**Supplementary Table S1. Organizations and URLs**

| **Organization (acronym)** | **URL** |
| --- | --- |
| African Registry of Assisted Reproduction (ANARA) | <https://anara-africa.com/> |
| American Society of Reproductive Medicine  (ASRM) | <https://www.asrm.org/> |
| American Urological Association  (AUA) | <https://www.auanet.org/> |
| Andrology Awareness EU | https://www.andrologyawareness.eu/ |
| Australian Men’s Shed Association | <https://mensshed.org/> |
| Centers for Disease Control and Prevention  (CDC) | <https://www.cdc.gov/art/artdata> |
| CONCEBIR | <https://www.concebir.org.ar/> |
| Contraception, Research and Development (CONRAD) | <https://www.conrad.org/> |
| COST Action ANDRONET | [https://www.andronet.cat/](https://urldefense.com/v3/__https:/www.andronet.cat/__;!!AQDxp81HYA!MWQ8aAb7XRVXYQo1wO1u_8JYzWrpPJGmXEhfhq3xu2tNHn_DjGfOn-PEWRvT4Yrc0VnWLiM3ewMUzlbugjZ-uDqWaOIBr8J2LVk$) |
| European Andrology Academy  (EAA) | <https://www.andrologyacademy.net/> |
| European Society of Andrological Urology (ESAU) | <https://uia.org/s/or/en/1100047845> |
| European Society for Human Reproduction and Embryology (ESHRE) | <https://www.eshre.eu/> |
| European Union (EU) | <https://european-union.europa.eu/institutions-law-budget/institutions-and-bodies/institutions-and-bodies-profiles_en> |
| Fertility Europe (FE) | <https://fertilityeurope.eu/european-atlas-of-fertility-treatment-policies/> |
| Fertility Society of Australia and New Zealand | <https://www.fertilitysociety.com.au/> |
| General Social Survey  (GSS) | <https://gssdataexplorer.norc.org> |
| Global Action on Men’s Health (GAMH) | <https://gamh.org/> |
| Global Web Index | [www.globalwebindex.com](http://www.globalwebindex.com) |
| Growsperm | <http://growsperm.eu> |
| Healthy Male | <https://www.healthymale.org.au/> |
| International Committee for Monitoring Assisted Reproduction Technologies (ICMART) | <https://www.icmartivf.org/> |
| International Male Infertility Genomics Consortium (IMIGC) | [www.imigc.org/](http://www.imigc.org/) |
| International Society of Andrology (ISA) | https://www.andrology.org/ |
| Latin American Registry of Assisted Reproduction (RLA) | <https://redlara.com/registro.asp> |
| Male Contraceptive Initiative (MCI) | <https://www.malecontraceptive.org> |
| International Male Infertility Genomics Consortium (IMIGC) | <http://www.imigc.org/> |
| Male Reproductive Health Initiative (*M*RHI) | <https://www.eshre.eu/Specialty-groups/Special-Interest-Groups/Andrology/MRHI> |
| Men’s Health Week | <https://www.gamh.org/mens-health-week/> |
| Movember | <https://es.movember.com/> |
| National Bureau of Statistics of China | <https://data.stats.gov.cn/english/> |
| National Institute of Child Health and Development (NICHD) | <https://www.nichd.nih.gov/> |
| Office on Women’s Health (OWH) | [www.womenshealth.gov](http://www.womenshealth.gov) |
| Organization of American States (OMS) | <https://www.oas.org/en/> |
| Pan American Health Organization (PAHO) | <https://www.paho.org/en> |
| Red Latinoamericana de Reproducción Asistida (REDLARA) | <https://redlara.com/> |
| Red TRAscender | <https://www.instagram.com/redtrascender/> |
| Resolve, The National Infertility Association | [www.resolve.org](http://www.resolve.org) |
| United Nations Department of Economic and Social Affairs Population Division | <https://population.un.org/wpp/Download/Standard/Population/> |
| UK’s National Survey of Sexual Attitudes and Lifestyles | <https://www.natsal.ac.uk> |
| USA census data | [www.data.census.gov](http://www.data.census.gov) |
