# Supplementary material for: Current global status of male reproductive health
Source: Hum Reprod Open. 2024 Apr 12;2024(2):hoae017. doi: 10.1093/hropen/hoae017 (PMC11065475; doi:10.1093/hropen/hoae017)
Supplement: hoae017_Supplementary_Table_S2 [file hoae017_supplementary_table_s2.docx]

**Supplementary Table S2. WHO publications**

| **Title** | **URL** |
| --- | --- |
| WHO definition of sexual health | <https://www.who.int/health-topics/sexual-health#tab=tab_2> |
| WHO definition of infertility | <https://www.who.int/news-room/fact-sheets/detail/infertility> |
| WHO laboratory manual for the examination and processing of human semen (6^th^ edition) (2021)  **ISBN:**978 92 4 0030787 | <https://www.who.int/publications/i/item/9789240030787> |
| Medical Eligibility Criteria for Contraceptive use (5^th^ edition)  **ISBN:**978 92 4 1549158 | <https://www.who.int/publications/i/item/9789241549158> |
| Selected Practice Recommendations for Contraceptive use (3^rd^ edition)  (2016)  **ISBN:**9789241565400 | <https://www.who.int/publications/i/item/9789241565400> |
| Ensuring Human Rights in the Provision of Contraceptive Information and Services  (2015)  **ISBN:**978 92 4 150674 8 | <https://www.who.int/publications/i/item/9789241506748> |
| Family Planning a Global Handbook for Providers (2018)  **ISBN:**978 0999203705 | <https://www.who.int/publications/i/item/9780999203705> |
