# Supplementary material for: Current global status of male reproductive health
Source: Hum Reprod Open. 2024 Apr 12;2024(2):hoae017. doi: 10.1093/hropen/hoae017 (PMC11065475; doi:10.1093/hropen/hoae017)
Supplement: hoae017_Supplementary_Table_S3 [file hoae017_supplementary_table_s3.docx]

**Supplementary Table S3.** Demographics and Global Analysis.

| **Region or Country included in the analysis**  **(Continent)** | **Area**  **(2021)^1^** | **Population**  **(thousands)**  **2022)^2^** | **Population density (people /km^2^)**  **(2020)^3^** | **Population age**  **0-14 / 15-64 (% of total population) (2022)^4^** | **Male Life expectancy at birth**  **(2021)^5^** | **Fertility rate**  **(births per woman) (2021)^6^** | **Gross Domestic Product (GDP) per capita in US Dollars**  **(2022)^7^** | **Current**  **Health Expenditure (% of GDP)**  **(year)^8^** |
| --- | --- | --- | --- | --- | --- | --- | --- | --- |
| Africa  (Sub-Saharan Africa) | 24,328,266  km^2^ | 1,211,170 | 49 | 42 / 55 | 58 | 4.6 | 1,690.4 | Egypt, Arab Rep: 4.36 (2020)  Nigeria: 3.38 (2020)  Ethiopia: 3.48 (2020) |
| Australia (Oceania) | 7,741,220  km^2^ | 25,979 | 3 | 18 / 65 | 81 | 1.7 | 64,491.4 | 10.65  (2020) |
| China  (People's Republic of China; PRC) (Asia) | 9,562,910  km^2^ | 1,412,175 | 150 | 17 / 69 | 75 | 1.2 | 12,720.2 | 5.59  (2020) |
| European Union  (Europe) | 4,254,350  km^2^ | 446,828^9^ | 112 | 15 / 64 | 78 | 1.5 | 37,149.6 | Germany:12.81 (2021)  France: 12.21 (2020) Italy: 9.45 (2021) |
| North America | 25,470,210  km^2^ | **372,281** | 21 | 18 / 65 | 74 | 1.6  (2021) | 74,164.7 | USA: 18.82 (2020) Canada: 11.68 (2021) |
| South America | 20,523,017  km^2^  (Latin America & Caribean) | 659,311  (Latin America & Caribean) | 33  (Latin America & Caribbean) | 23 / 68  (Latin America & Caribbean) | 69  (Latin America & Caribbean) | 1.9  (Latin America & Caribbean) | 9,474.5  (Latin America & Caribbean) | Brazil: 10.31 (2020)  Argentina: 9.98 (2020)  Colombia: 8.99 (2020)  Mexico: 6.24 (2020) |

Abbreviation: sq mi: square miles

1. <https://data.worldbank.org/indicator/AG.SRF.TOTL.K2>
2. <https://databankfiles.worldbank.org/public/ddpext_download/POP.pdf>
3. <https://data.worldbank.org/indicator/EN.POP.DNST>
4. <https://data.worldbank.org/indicator/SP.POP.0014.TO.ZS>
5. <https://data.worldbank.org/indicator/SP.DYN.LE00.IN>
6. <https://data.worldbank.org/indicator/SP.DYN.TFRT.IN?locations=ZG>
7. <https://data.worldbank.org/indicator/NY.GDP.PCAP.CD> GDP /capita: A country's Gross Domestic Product (GDP) divided by its total population.
8. <https://data.worldbank.org/indicator/SH.XPD.CHEX.GD.ZS> (when countries listed, they were selected by largest population in the region)

9) ["Eurostat – Population on 1 January 2022"](https://ec.europa.eu/eurostat/databrowser/view/TPS00001/bookmark/table?lang=en&bookmarkId=6ef61f16-dadc-42b1-a6ce-3ddfda4727e8). Retrieved 9 February 2023.
